# Subjective Freedom of Speech: Why Do Citizens Think They Cannot Speak Freely?

**DOI:** 10.1007/s11615-022-00414-6

**Published:** 2022-08-11

**Authors:** Jan Menzner, Richard Traunmüller

**Affiliations:** University of Mannheim, Mannheim, Germany

**Keywords:** Freedom of speech, Self-censorship, Cancel culture, Populism, German political culture, Freedom of expression, Meinungsfreiheit, Selbstzensur, Cancel Culture, Populismus, Politische Kultur, Redefreiheit

## Abstract

We provide the first systematic research into the origins of subjective freedom of speech in Germany. Relying on the GLES 2021 Cross-Section Pre-Election Survey, which includes a newly designed survey item on subjective freedom of speech, we evaluate a whole range of plausible candidate hypotheses. First, we contribute to cumulative research by testing the explanatory factors in Gibson (1993)—citizens’ social class, their political involvement and political preferences, and their personality dispositions—for the German case. Second, we move beyond the state of the art and test three new hypotheses that reflect more recent political developments and arguments in the free speech debate: the role of social media, increasing political and social polarization, and the rise of populism. Importantly, all hypothesis tests reported in this paper have been preregistered prior to data collection. Our results reveal that three explanatory factors are significantly, consistently, and substantively related to subjective free speech in Germany: political preferences, populist attitudes, and identification with the Alternative for Germany party.

**Supplementary Information:**

The online version of this article (10.1007/s11615-022-00414-6) contains supplementary material, which is available to authorized users.

## Abstract (de)

Wir liefern die erste systematische Untersuchung über die Ursprünge der subjektiven Meinungsfreiheit in Deutschland. Basierend auf der Vorwahlerhebung des GLES Querschnitts 2021, die ein neu entworfenes Umfrage-Item zur subjektiven Meinungsfreiheit enthält, evaluieren wir eine ganze Reihe von plausiblen Hypothesen. Erstens tragen wir zur kumulativen Forschung bei, indem wir die Erklärungsfaktoren von Gibson (1993) – soziale Schichtzugehörigkeit der Bürger, ihr politisches Engagement und ihre politischen Präferenzen sowie ihre Persönlichkeitsdispositionen – für den deutschen Fall testen. Zweitens gehen wir über den Stand der Forschung hinaus und testen drei neue Hypothesen, die neuere politische Entwicklungen und Argumente in der Debatte um Meinungsfreiheit widerspiegeln: die Rolle der sozialen Medien, die zunehmende politische und soziale Polarisierung und der Aufstieg des Populismus. Alle Analyseschritte und Hypothesentests der vorliegenden Studie wurden vor der Datenerhebung präregistriert. Unsere Ergebnisse zeigen, dass drei Erklärungsfaktoren signifikant, konsistent und substanziell mit der subjektiven Meinungsfreiheit in Deutschland zusammenhängen: politische Präferenzen, populistische Einstellungen und die Identifikation mit der AfD.

## Introduction

Restrictions on free expression have increased significantly in recent years and are among the most troubling indicators of “democratic backsliding” in liberal democracies (Mechkova et al. 2017; V‑Dem Institute 2021). But even in stable liberal democracies like Germany, where freedom of speech is guaranteed and common indicators of political freedom show little decline,^1^ growing segments of the population express the subjective feeling that they are no longer free to speak their mind. Whereas in 1971 some 83% of the German population felt they could “freely express their political opinion,” 50 years later in 2021 this share had shrunk to a mere 45% (IfD Allensbach, cf. Petersen 2021).^2^ This poses an important research puzzle: Why do these citizens think they cannot speak freely?

Some indications may be gleaned from the many polarized public debates that revolve around free speech and its limits. These include discussions over the spread of hate speech and misinformation online but also concern a perceived lack of viewpoint diversity in mainstream media outlets (e.g., in the wake of the 2015 refugee crisis or, more recently, the COVID-19 pandemic), the regulation of online speech by internet platform providers (e.g., via the Network Enforcement Act, or NetzDG), the rise of a so-called cancel culture in higher education and other cultural institutions on one hand and of strategic right-wing populist narratives of a free speech crisis on the other, and, finally, the sense and sensibilities of “nondiscriminatory” and “gender-inclusive” speech norms. Whereas one side in the debate bemoans an increasingly restrictive and moralistic discursive climate that threatens free speech in Germany, the other side either dismisses this perception as hysteria or even welcomes changing speech norms to fight what it views as outdated privileges and discrimination.

Against the background of these debates, it is curious that political science has not paid more attention to the question of whether and why citizens think they can express themselves freely. While recent work has begun to investigate “campus cancel culture” (e.g., Revers and Traunmüller 2020; Traunmüller 2022; Norris 2021; Kaufmann 2021) and has looked into the democratic effects of hate speech regulation (e.g., Van Spanje and De Vreese 2015, Jacobs and Van Spanje 2021) the more fundamental question of what explains citizens’ subjective freedom of speech remains largely unanswered (but see Gibson 1992, 1993 and Gibson and Sutherland 2020 for the United States).

Our goal is to fill this gap and to provide the first systematic piece of research into the origins of subjective freedom of speech in the interesting case of Germany. Due to their historic experience with two dictatorships in the last century, Germans are arguably highly sensitive to both the limits and the value of free speech. The atrocities committed under the Nazi regime have led to the conviction that freedom does not trump equal dignity and to respective laws that exclude racist and antisemitic hate speech from protected speech (Bleich 2011). At the same time, many eastern Germans have lived under the German Democratic Republic regime and therefore experienced state repression and censorship firsthand, which may make them particularly sensitive to any encroachment on their freedom of speech. But apart from these idiosyncrasies, Germany shares many of the free speech challenges of other liberal democracies that result from growing cultural diversity, expanding communication technology, and the associated political conflicts.

Understanding citizens’ perceptions of free speech is important for at least two reasons. For one, they may in fact be accurate (especially if citizens are highly educated and well informed, cf. Guriev and Treisman 2019). The perceptions may therefore tell us something about the quality of a democracy that would otherwise be hard to observe. But subjective freedom of speech is also relevant on a deeper, intrinsic level and even if citizens’ perceptions are inaccurate. According to Dahl (1971), democracy requires that all citizens have the unimpaired opportunity to profess their preferences. This requirement rests on citizens’ subjective sense that they are in fact free to express themselves.^3^

In our initial attempt to find answers to the question of why citizens think they cannot speak freely, we rely on the *GLES 2021 Cross-Section Pre-Election Surve*y, which includes a newly designed survey item on subjective freedom of speech that explicitly taps into public expression (“People like me are no longer allowed to express their opinions freely in public”). We deliberately cast a wide empirical net and evaluate a whole range of plausible candidate hypotheses. First, we “replicate” Gibson (1993) as closely as possible. To the best of our knowledge, this is the most comprehensive previous study into the topic. We take this study as point of departure to ground our work and to contribute to cumulative research by testing the proposed explanatory factors—citizens’ social class, their political involvement and political preferences, and their personality dispositions—using novel data for the German case.

Second, we move beyond the state of the art and test three new hypotheses that reflect more recent political developments and arguments in the free speech debate: the role of social media, increasing political and social polarization, and the rise of populism. While we are not in the position to present a longitudinal research design, we propose that to understand the apparent *decline* of subjective freedom of speech, one must consider explanatory factors that reflect actual change in political circumstances.

All planned analyses and hypothesis tests on subjective freedom of speech reported in this paper were preregistered prior to data collection.^4^ The preregistration fully documents all tested hypotheses regardless of their eventual merit. This way, we avoid the problem of confounding the processes of hypothesis generating and hypothesis testing (Nosek et al. 2018) and the problem of publication bias in favor of statistically significant results (Franco et al. 2014). In addition, we restrict and make fully transparent our own researcher degrees of freedom, including our choice of variables, coding schemes, and model specifications (Simmons et al. 2011).

Importantly, our preregistration guards against accusations of politically motivated results in what has become a topic of highly polarized debate (cf. Clark and Winegard 2020). By preregistering the analysis (and the editors’ preacceptance of our plan), we commit ourselves to the results regardless of our own political preferences. As researchers we are ordinary humans and thus politically motivated information processors who look for results that support our political ideas and avoid results that contradict them (Taber and Lodge 2006; Kahan 2012). To guard against this bias, we supplement the preregistration with the emerging open science practice of “adversarial collaboration” (Clark and Tetlock 2021): The two authors of this paper not only differ in their political ideology but also in their views on the problem of subjective freedom of speech.^5^ By working together, we hope to keep each other’s political biases in check and produce more credible results.^6^

## What Is Subjective Freedom of Speech?

While subjective freedom of speech has not received much scholarly attention, a central variable in the study of authoritarian politics is the closely related concept of self-censorship (Hayes et al. 2006; Kuran 1991, 1997; Jiang and Yang 2016; Bar-Tal 2017; Shen and Truex 2021). Self-censorship refers to the strategic behavior of withholding one’s true opinion to avoid negative consequences. It occurs in both private and public contexts and under both authoritarian and democratic regimes. Although self-censorship is not necessarily a democratic bad, it is linked to several undesirable consequences, such as inhibited political change and the persistence of unpopular policies, distorted public knowledge, and impoverished public debates, as well as the occurrence of completely unexpected and potentially disruptive political events (Kuran 1997).

While conceptually distinct,^7^ understanding why individuals self-censor sheds important insights into the nature of subjective freedom of speech. Because self-censorship is a strategic behavior, it is useful to model it as being based on a simple cost–benefit calculus, in which an individual would choose to express themselves if their expected benefits of doing so exceed the associated costs. Following Ong (2021), we specify a simple expected utility model as

1 $$\text{EU}_\text{express}=\text{B}_\text{express}-\text{C}_\text{express}-(\text{p}_\text{sanction}\,\text{C}_\text{sanction})$$

where EU_express_ is the expected utility associated with expressing one’s views, B_express_ is the expected benefits from this expression (e.g., a gain in reputation from peers or psychological gratification derived from authentic expression), and C_express_ refers to the cost of expressing oneself (e.g., required knowledge or a costly medium for publishing one’s views). In addition, p_sanction_ represents the probability of an individual to encounter “sanctions” for their expression, and C_sanction_ is the cost associated with these sanctions.

The model thus follows a social scientific conception of freedom of speech that differs from the traditional legalistic understanding of free speech as a constitutionally guaranteed “basic right” that either “exists or not.” Instead, the *degree* of freedom of expression is determined by the extent to which it is regulated, or in other words, by *how much costs are imposed* on free expression. *Subjective* freedom of speech is thus simply an individual’s *perceived *costs (C_express_) associated with expressing what they believe, their *perception* of how likely it is that they will be sanctioned for it (p_sanction_), and their *expected *costs when being sanctioned for expressing their views (C_sanction_). In short, subjective freedom of speech does not result from a cost–benefit calculus but *is* the cost–benefit analysis itself. For those who lack subjective freedom of speech, the perceived costs of speaking out are just too high.

## What Drives the Subjective Costs of Free Expression?

In the following we reconstruct the theoretical arguments proposed in Gibson (1993) with reference to the behavioral model introduced in the previous section and derive several testable hypotheses.^8^

### Social Class

Starting with social class, the basic rationale put forward by Gibson (1993) is that individuals who are high in social status, have more resources at their disposal, and have more prestigious occupations are less dependent on and vulnerable to the constraints on their free speech. Or to put it in terms of the behavioral model, individuals high in social class are less likely to be sanctioned (p_sanction_) and/or the sanctions are less costly to them (C_sanction_). Even though Gibson (1993) does not find significant empirical evidence, we follow the suggested direction of the hypothesis in our replication.^9^

#### H_1_

Individuals who rank themselves lower in social class are more likely to think they cannot speak freely.

### Political Involvement

Next, Gibson (1993, p. 127) hypothesized that citizens with higher political involvement are also more likely to perceive greater freedom and found some evidence that greater political interest and political knowledge were related to less self-censorship. To argue in terms of our behavioral model, individuals who are less interested in politics, have less knowledge of political processes, and possess less internal political efficacy may face higher subjective costs (C_express_) for free expression because it takes time, skill, and effort to form and effectively articulate political beliefs. In addition, those less involved in politics may not know about or have access to channels for expressing their views. Of course, one could also argue that increased political involvement might also expose people to more conflict and perceived pushback on their opinions, suggesting higher levels of perceived p_sanction_.

#### H_2_

Individuals with lower political involvement are more likely to think they cannot speak freely.

### Political Preferences

Next to general political involvement, Gibson argues that the *content* of citizens’ political preferences and beliefs may be consequential for their perceptions of freedom (Gibson 1993, p. 128). Specifically, he finds that those who have a greater preference for social order over liberty are also more likely to perceive governmental constraints and more likely to engage in self-censorship. Furthermore, those who are more politically tolerant also perceive more subjective political freedom. One could transpose this “projection argument” into the ideological left–right continuum by roughly equating the conservative right with a preference for social order and the progressive left with political tolerance. But a stream of recent research suggests that equating only one political side with political (in)tolerance is empirically untenable (Crawford and Pilanski 2014; Revers and Traunmüller 2020).

More likely, therefore, conservative or right-leaning citizens will perceive a higher probability of “sanctions” (p_sanction_) because their political views increasingly clash with the changing cultural norms that are part of the “silent revolution,” i.e., a broad cultural shift toward more progressive, social–liberal, and postmaterialist values that embrace multiculturalism, immigration, gender equality, and sexual diversity (Inglehart and Welzel 2005; Norris and Inglehart 2019). The perception of a subjective lack of freedom of speech is further enhanced by the introduction of new “politically correct” speech norms endorsed by the progressive left (e.g., so-called gender-inclusive language or the banning of racist or sexist expressions) and increasingly adapted by organizations such as universities and the public media (Campbell and Manning 2018). Thus, conservative citizens may feel they are not only sanctioned for the *content* of their beliefs but also for *the way* they express them.

#### H_3_

Individuals on the political right are more likely to think they cannot speak freely, and this effect will be stronger for the cultural than for the economic political preference dimension.

An interesting variation of this argument is provided by Loury (1994). According to his theory, it is precisely the individuals who actually *want *to be part of the community and share its values who perceive the highest subjective costs of being sanctioned (C_sanction_). For true deviants, on the other hand, these subjective costs are negligible (Loury 1994, p. 437).

#### H_4_

Individuals with moderate political views are more likely to think they cannot speak freely, suggesting an inverse U‑shaped relationship between political ideology and subjective free speech.

### Personality

Finally, the perceived cost of free expression and associated sanctions (C_express_, C_sanction_) may differ across citizens’ psychological personality traits. According to Gibson (1993, p. 128), the “internal psychological makeup” of a person could have just as much effect on their perception of freedom as external factors. He proposes that “closed-minded” individuals tend to see the political world in terms of good and evil and thus perceive more restrictions to their freedom by their opponents (Gibson 1993, p. 128). In addition, and since every expression of political views comes with the risk of disagreement and rejection, individuals with “low self-esteem” should perceive higher costs of free expression. We test the merit of Gibson’s argument using two of the “Big Five” personality traits: neuroticism and openness to experience (Mondak 2010).^10^

#### H_5_

Individuals with lower levels of openness to experience are more likely to think they cannot speak freely.

#### H_6_

Individuals with higher levels of neuroticism are more likely to think they cannot speak freely.

## Some More Recent Sources of Subjective Freedom of Speech

Since the key stylized fact that motivates the present research is an apparent *decline* in subjective freedom of speech over the last decades (Gibson and Sutherland 2020; Petersen 2021), we next consider three fundamental changes in the political conditions that may account for this decrease and attempt a “microfoundation” that casts these explanatory factors in terms of perceived individual costs.

### Social Media

One potentially important factor in explaining subjective freedom of speech that was not yet on the agenda in the 1990s is the new opportunity afforded by drastic changes in communication technology. The most notable manifestation of this change is the advent of social media platforms such as Facebook, Twitter, YouTube, Instagram, and the like. Social media has an ambivalent connection to the question of free speech (King et al. 2013; Tucker et al. 2017; Roberts 2018, 2020). On the one hand, social media can be viewed as a democratizing technology that allows everyone who has access to the Internet to freely share their opinion with virtually millions of other users around the globe. Social media has thus drastically cut the costs of free expression, C_express_ (Ong 2021). On the other hand, the initial enthusiasm about the democratic potential of social media has considerably cooled down in recent years. Concerns over the spread of misinformation, “fake news,” and “hate speech” have prompted both state governments and social media platform providers to increase the regulation of online speech. In effect, freedom of expression online is now more restricted than it used to be. The more an individual uses social media, the higher the chances they will perceive these restrictions, i.e., the greater p_sanction_ and C_sanction_ (Ong 2021). Of course, p_sanction_ and C_sanction_ are not restricted to formal legislation or platform regulations but may also relate to informal social mechanisms and costs induced by other users. One specific concern with social media is that it facilitates a hostile environment in which users passively observe or are even themselves subjected to insults, abuse, and threats by other users when voicing their opinion. Because these reactions (at least to some extent) presuppose the formulation of opinions, we also argue that the more outspoken an individual is online, the higher the probability of potentially negative societal feedback or sanctions (p_sanction_).

#### H_7_

Individuals with higher social media usage are more likely to think they cannot speak freely.

### Political and Social Polarization

An alternative, yet related explanation for a perceived lack of freedom of speech focuses on political and social polarization (Gibson and Sutherland 2020). Citizens in several liberal democracies are increasingly divided along partisan or political lines, where a heightened “in-group” identification coincides with a pronounced dislike and open rejection of members of political “out-groups” (Iyengar and Westwood 2015; Westwood et al. 2018). There are two mechanisms that explain why political and social polarization may reduce subjective freedom of speech. The first is that expressing one’s true opinions is less rewarding (lower B_express_) or even stressful (higher C_express_) in a highly polarized environment in which people not only hold opposing beliefs but may actively dislike each other (Hayes et al. 2006; Gibson and Sutherland 2020). The second mechanism relates to so-called echo chambers or filter bubbles. As a result of political polarization, individuals increasingly live and socialize with people who are similar to themselves and share the same political views (Huber and Malhotra 2017). Since politically homogeneous social networks are more likely to exert conformity pressures and to sanction deviant opinions, free expression will be perceived as more costly (higher p_sanction_ and C_sanction_). Conversely, a wide range of perspectives and ideas within one’s social network is evidence that different political viewpoints are not only possible and tolerated but even legitimate and welcome (Gibson 1992; Mutz 2002).

#### H_8_

Individuals with less diverse discussion networks are more likely to think they cannot speak freely.

### Populism

Finally, limited freedom of speech is also a regular ingredient of so-called populist narratives, especially those put forward by parties of the far right, which have gained political prominence in many liberal democracies (Moffitt 2017). Populism generally refers to a “thin” ideology that views society as deeply divided between “the pure people” and “the corrupt elite” and that postulates that politics should be the exclusive expression of the unison “general will” of the people (Mudde 2004; Akkerman et al. 2014; Schulz et al. 2018). Because this imagined “general will” is a core element of populism, it is logically opposed to all limits placed on its public expression (Mudde 2004). Furthermore, due to the inherent anti-elitism, populist narratives describe political elites as uninterested in the public’s opinion or even actively opposed to it. This described opposition can, for example, take the form of furthering discourse norms such as “political correctness,” which is seen as censoring the alleged majority in favor of a minority (Brubaker 2017). In short, citizens with higher affinity to populist attitudes should be more likely to subscribe to the idea that free expression is stifled by the elites and to perceive a higher probability and cost of sanctions (p_sanction_ and C_sanction_).

#### H_9_

Individuals with a higher affinity to populism are more likely to think they cannot speak freely.

An alternative mechanism that links populist narratives to subjective freedom of speech runs via party cues. There is ample evidence that voters form their opinions and beliefs based on party cues (e.g., Cohen 2003; Brader and Tucker 2012; Slothuus and Bisgaard 2021). Party manifestos and press releases (Breeze 2019; Goerres et al. 2018; Keil 2020) as well as prominent politicians of the Alternative for Germany party (AfD) actively push a narrative of limited freedom of speech in Germany. For example, Bundestag group leader Alexander Gauland stated that people who articulate “uncomfortable truths and not elite-pleasing opinions publicly […] have to reckon with social annihilation” (AfD 2018). Further, many election posters or campaign appeals contain demands to defend freedom of speech (SWR 2019; AfD 2020). Thus, we expect that identification with the AfD is associated with adoption of the party’s narrative that the probability and costs of sanctions (p_sanction_ and C_sanction_) for voicing nonmainstream opinions are high.

#### H_10_

Individuals who identify with the AfD are more likely to think they cannot speak freely.

## Data and Methods^11^

### Data

For our analyses we rely on the *GLES 2021 Cross-Section Pre-Election Surve*y (GLES 2022), which was fielded between 26 August and 25 September 2021. Using a multistage register sample, *N* = 5116 respondents were sampled from German citizens aged 16 years or older and living in private households. Data were collected using a mixed-mode design including both computer-assisted web-based interviews and paper-and-pencil interviews. Interview participation was incentivized with 5 euros paid unconditionally before the interview.

### Subjective Freedom of Speech

The variable of main interest to our study is a newly designed survey item of subjective freedom of speech: “People like me are no longer allowed to express their opinions freely in public.” It is part of an item battery including a total of four items on feelings of marginalization. Respondents are asked to rate their agreement on a 5-point scale, which we reversed such that higher levels of agreement indicated a greater lack of subjective freedom of speech. This new item circumvents some of the problems and ambiguities surrounding the validity of previous question wordings.^12^

### Explanatory Variables

Here, we only briefly discuss the explanatory variables used in our analyses. We refer to Tables A.2.1–A.2.4 in the online appendix for questionnaire variable numbers, exact question wordings, and answer scales, as well as more detailed coding decisions. Descriptive statistics, including number of observations, missing values, means, standard deviations, and minimum and maximum variable values, are included in Table A.3.0 in the online appendix. Unless stated otherwise, we treat ordered categorical variables or count variables as quasicontinuous and assume linear effects.

To explain subjective freedom of speech, we operationalize *social class* using a subjective measure, asking respondents to identify which out of six social classes they feel they belong to. *Political involvement* is measured using a total of three variables: respondents’ general political interest, political knowledge (binary), and perceived political efficacy. To tap into respondents’ *political preferences*, we rely on the standard 11-point ideological left–right scale. We enter this variable both linearly and with an additional quadratic term. For more nuance in measuring political preferences, we also distinguish between the economic and the cultural dimension of the political preference space. To measure respondents’ *personality*, we rely on a Big Five short scale. Openness to experience is measured by asking respondents whether they are imaginative and whether they have artistic interests. Neuroticism is measured with two items eliciting respondents’ ability to handle stress and their tendency to get nervous. We construct simple mean scores for each personality dimension. *Social media use *is operationalized threefold: First, respondents are asked on how many days a week they use the internet. Second, they can indicate which social media platforms they use (out of a list of 14 different platforms, including Facebook, Twitter, Instagram, etc.), which we aggregate into a count variable. Third, respondents are asked whether they partake in varying forms of political participation online, with the first three subitems directly relating to social media posting, sharing, and liking of political content, respectively. *Political and social polarization* is operationalized by the degree of opinion diversity in respondents’ discussion networks. This measure is based on the frequency of disagreement between respondents and their two most important political discussion partners. Both answers are averaged, and respondents with no political discussion partner are assigned the lowest score. Respondents’ affinity to *populism* is captured using a scale consisting of a total of six items tapping into several relevant aspects of populism, such as cynicism toward political elites and preferences for popular sovereignty. To measure identification with the AfD, we rely on a binary item about party identification.

### Control Variables

For the models explaining subjective freedom of speech, we control for several key sociodemographics that may be related to both subjective freedom of speech and the hypothesized explanatory variables (and which have also been used for constructing survey weights). Specifically, we include respondents’ gender, age, and education, along with a simple dummy for respondents from eastern parts of Germany. In addition, we include respondents’ migration background and whether they live in a rural or urban environment.

### Data Exclusion and Missing Data

We generally treat “don’t knows” as missing values (for exceptions, see Tables A.2.1–A.2.4 in the online appendix). We then employ multiple imputation (M = 5) using chained equations (Van Buuren 2007), run our models on all five imputed data sets, and present the combined results (Little and Rubin 1987).

### Sampling Weights

We include all variables used for the weighting as control variables in our regression models to adjust for any sample imbalances (see Gelman 2007).

## Analysis Plan^13^

### Statistical Models

We rely on ordered logit models as our default modeling choice. We start out with a model that includes only the control variables to get an impression of the sociodemographic distribution of the perception that one cannot speak freely (see Table A.3.1 in the online appendix). In a next step, we estimate models for each hypothesis *separately*, each time including the respective explanatory variable(s) along with the controls (M1–M11; see Table A.3.2 in the online appendix). The final model specifications are joint models including either subsets (M12–M14; Table 1) or all (M15–M16; Table 2) explanatory variables along with the controls.

**Table 1:** Submodels: explanations of subjective freedom of speech (ordered logistic regression)

		M12			M13			M14	
	Est	SE	q	Est	SE	q	Est	SE	q
*Social class*									
Subjective social class	−0.21	0.03	0.00*	−0.20	0.03	0.00*	–	–	–
*Political involvement*									
Political interest	0.07	0.04	0.11	0.06	0.04	0.18	–	–	–
Political knowledge	−0.21	0.06	0.00*	−0.19	0.06	0.00*	–	–	–
Political efficacy	−0.13	0.03	0.00*	−0.13	0.03	0.00*	–	–	–
*Political preferences*									
Left–right ideology	0.06	0.02	0.00*	0.05	0.06	0.49	–	–	–
Economic preferences	0.12	0.01	0.00*	0.06	0.05	0.37	–	–	–
Cultural preferences	0.24	0.01	0.00*	−0.01	0.05	0.93	–	–	–
Left–right ideology squared	–	–	–	0.00	0.01	0.86	–	–	–
Economic preferences squared	–	–	–	0.01	0.00	0.29	–	–	–
Cultural preferences squared	–	–	–	0.02	0.00	0.00*	–	–	–
*Personality*									
Openness to experience	−0.01	0.03	0.90	−0.02	0.03	0.66	–	–	–
Neuroticism	0.05	0.04	0.25	0.06	0.04	0.17	–	–	–
*Social media*									
Frequency of internet use	–	–	–	–	–	–	−0.02	0.01	0.20
Social media platforms	–	–	–	–	–	–	−0.02	0.02	0.35
Political participation online	–	–	–	–	–	–	0.00	0.04	0.97
*Social and political polarization*									
Social network diversity	–	–	–	–	–	–	−0.08	0.03	0.01*
*Populism*									
Populism index	–	–	–	–	–	–	1.07	0.04	0.00*
AfD identification	–	–	–	–	–	–	1.82	0.13	0.00*
*Sociodemographic controls*	–	*Yes*	–	–	*Yes*	–	–	*Yes*	–

*q-values < 0.1 (i.e., corrected *p*-values that allow for a false discovery rate among significant findings of maximum 10%)

*AfD* Alternative for Germany party, *Est* estimate, *SE* standard error

**Table 2:** Main results: explanations of subjective freedom of speech (ordered logistic regression)

		M15			M16	
	Est	SE	q	Est	SE	q
*Social class*						
Subjective social class	−0.11	0.03	0.00*	−0.11	0.03	0.00*
*Political involvement*						
Political interest	0.05	0.04	0.30	0.05	0.04	0.29
Political knowledge	−0.13	0.06	0.06*	−0.13	0.06	0.06*
Political efficacy	−0.07	0.03	0.08*	−0.07	0.03	0.07*
*Political preferences*						
Left–right ideology	0.06	0.02	0.00*	0.12	0.06	0.11
Economic preferences	0.11	0.01	0.00*	0.10	0.06	0.12
Cultural preferences	0.17	0.01	0.00*	0.12	0.05	0.04*
Left–right ideology squared	–	–	–	0.00	0.01	0.42
Economic preferences squared	–	–	–	0.00	0.00	0.96
Cultural preferences squared	–	–	–	0.00	0.00	0.35
*Personality*						
Openness to experience	−0.03	0.03	0.36	−0.04	0.03	0.35
Neuroticism	0.08	0.04	0.06*	0.08	0.04	0.06*
*Social media*						
Frequency of internet use	0.00	0.01	0.78	−0.01	0.01	0.77
Social media platforms	−0.01	0.02	0.66	−0.01	0.02	0.66
Political participation online	0.07	0.04	0.13	0.07	0.04	0.13
*Social and political polarization*						
Social network diversity	−0.05	0.03	0.11	−0.05	0.03	0.12
*Populism*						
Populism index	0.85	0.05	0.00*	0.85	0.05	0.00*
AfD identification	1.24	0.14	0.00*	1.23	0.14	0.00*
*Sociodemographic controls*	*Yes*	–	–	*Yes*	–	–

* *q*-values < 0.1 (i.e., corrected *p*-values that allow for a false discovery rate among significant findings of maximum 10%)

*AfD* Alternative for Germany party, *Est* estimate, *SE* standard error

### Inference Criteria

For statistical inference, we rely on classical frequentist *p*-values. However, given the multitude of hypotheses we plan to test, we heed the multiple comparisons problem and correct these *p*-values to guard against false discoveries. Specifically, we rely on the Benjamini and Hochberg (1995) procedure to control the false discovery rate, i.e., the expected proportion of false discoveries among all significant hypotheses.^14^ In defining the first “family” of tests, we count all tests for explanatory variables (not the control variables) in the model specifications M1–M16. Each set of robustness tests is treated as its own “family” of tests. We judge hypotheses as having survived if a) at least one of its respective indicators reaches statistical significance in b) the full model specification M16 and c) remains robust under the two alternative specifications suggested as robustness tests (see below).

### Reliability and Robustness Testing

In our robustness tests we first focus on our key variable, subjective freedom of speech, and gauge how our inferences change when we alter its assumed measurement scale. While by default we model the subjective freedom of speech survey item as an ordered categorical outcome, we check the robustness of our findings when treating it a) as quasicontinuous in a linear regression model and b) as a binary variable in a logistic regression model (see Table A.3.3 in the online appendix). In the latter case, we join the “agree” and “strongly agree” categories and contrast them with the remaining three categories. For these robustness tests, we rerun only model specification M16. Given the high number of hypotheses, we apply a very strict robustness criterion and only report a given result as “robust” when it reaches statistical significance in all three alternative specifications. For a second, more substantively motivated, robustness test, we run separate specifications of M16 for East and West (omitting the East–West dummy, of course; see Table A.3.4 in the online appendix).^15^ Appendix A1 further discusses our considerations regarding effect sizes, statistical power, and potential issues with violated assumptions and model nonconvergence.

## Results

### Description: A First Look at Subjective Freedom of Speech

**Fig. 1:**
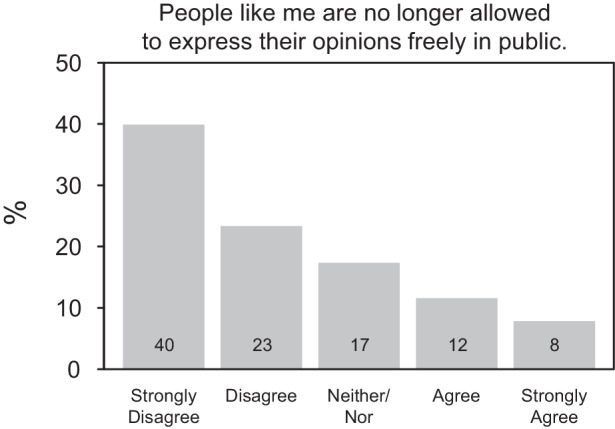
Distribution of subjective freedom of speech. Survey weighted to adjust for sex, age, education level, and residence in eastern or western Germany

Figure 1 shows the (weighted) distribution of the subjective freedom of speech survey item. Most of the German population feel free to speak their mind in public. Some 23% of respondents disagree and 40% even strongly disagree with the statement that “people like them” are no longer allowed to freely express their opinions. Yet at the same time, one in five persons agree with this statement (8% of whom agree strongly) and report a lack of a subjective freedom of speech. The remaining 17% are undecided in their evaluation of this key civil liberty.

Table A.3.1 in the online appendix further shows that subjective freedom of speech is not distributed evenly within the German population. Although there are virtually no gender differences, and most age groups exhibit similar levels of subjective freedom of speech (except for the 30–44-year-olds, who feel less free), we find that higher education and living in more urban areas is, on average, positively associated with subjective freedom of speech. In addition, eastern Germans and migrants tend to have lower levels of subjective freedom of speech. Taken together, this suggests that a lack of subjective freedom of speech may be an indicator of political grievance among segments of society that are, on average, less privileged. Of course, the grievances potentially vary in origin and strength between those groups. Thus, we seek to understand what drives this sentiment and will account for these sociodemographic differences in the following analyses.

### Main Results: Explaining Subjective Freedom of Speech

In our attempt to explain the lack of subjective freedom of speech, we start out by looking at *separate* models for each hypothesized factor (while controlling for sociodemographics; see Table A.3.2 in the online appendix). All explanations originally proposed by Gibson (1993) have at least some merit in the German context. Higher *social class* is significantly related to more subjective freedom of speech (M1). All three dimensions of *political involvement*—greater political interest (M2), political knowledge (M3) and political efficacy (M4)—are also significantly associated with more subjective freedom. In terms of *political preferences*, those on the right of the ideological spectrum are more likely to say they cannot speak freely, and this holds for the economic and even more strongly for the cultural preference dimensions (M5). However, we find no evidence for an inverse U‑shape that would suggest that political moderates are the ones most likely to feel unfree to express themselves (M6). The psychological* personality trait *of openness is related to more subjective free speech (M7), but neuroticism does not have an effect (M8).

To evaluate how these explanations for subjective freedom of speech interrelate and how they perform when tested against each other, a first *joint* model simultaneously includes all variables proposed by Gibson (1993) (while controlling for sociodemographics; M12 in Table 1). The negative relation between subjective social class and the perceived freedom to speak remains significant when controlling for differences in political involvement, political preferences, and personality traits. Individuals who place themselves on the right of the political spectrum and who have conservative economic and cultural beliefs are more likely to feel unfree to speak, net of their socioeconomic status, political involvement, and personality. Again, there is no sign of a curvilinear relation between political ideology and subjective freedom of speech (M13, Table 1). Greater political involvement is still associated with greater subjective freedom of speech when taking into consideration class, preferences, and personality. However, only political knowledge and efficacy remain significantly associated with an increase in perceived freedom, while political interest loses its significance. Personality loses its predictive power once we account for social class, political involvement, and political attitudes.

Our three new hypotheses provide additional insights into the drivers of subjective freedom of speech, at least when viewed in separation. When it comes to the role of *social media*, the theoretical ambiguity about its relation to free speech is reflected in mixed results. While frequent internet use is generally associated with more subjective free speech, active political online participation is related to less subjective freedom of speech, and the number of social media platforms a respondent uses is not associated at all (M9). Less polarized respondents with more politically diverse social networks are also less likely to say they are not allowed to speak freely (M10). Finally, those who hold populist views and those who identify with the AfD are more likely to feel excluded from speaking publicly (M11).^16^ Since social media, polarization, and populism are often viewed as tightly coupled phenomena in the current political environment, we also include these three explanatory factors in a joint model (while controlling for sociodemographics; see M14 in Table 1). Interestingly, once we account for the polarization or diversity of individuals’ discussion networks and their affinity to populist attitudes and party identification, none of the social media use variables explains subjective freedom of speech anymore. In the same model, being exposed to more diverse opinions in one’s own social networks is still related to more perceived freedom to speak. Lastly, populist attitudes and identification with the AfD remain significantly and strongly associated with decreases in the subjective sense of being able to speak freely, regardless of social media use or network diversity.

As laid out in the analysis plan, we base our *decisive* hypothesis tests on the final model specification, which integrates *all* the explanatory variables discussed above in one large joint model (see M15 and M16 in Table 2, where M16 includes the squared ideology variables proposed in hypothesis H4). Using this criterion, we find support for most of the hypotheses inspired by Gibson (1993): Individuals who rank themselves lower in social class (H1), who are on the right of the political spectrum, both economically and culturally (H3), and who are high in neuroticism (H6) are more likely to think they are not allowed to speak freely. Regarding political involvement (H2), the model shows a similar pattern as discussed before. Individuals with lower levels of political knowledge and efficacy (but not political interest) are significantly more likely to think they are not allowed to speak. The evidence contradicts the notion that the politically moderate (H4) and those low on openness to experience (H5) lack subjective freedom of speech. Regarding the more contemporary political hypotheses that we introduced, we find that individuals with a higher affinity to populism (H9) and those who identify with the AfD (H10) are more likely to feel unfree to speak their minds. However, neither active and extensive social media use (H7) nor more politically diverse social networks (H8) seem to be significant drivers of subjective freedom of speech or a lack thereof.

Because of the multitude of hypotheses tests, we also impose a strict robustness requirement. Only three hypothesized explanatory factors fully survive this criterion and prove to be completely robust explanations of subjective freedom of speech: political preferences, populist attitudes, and identification with the AfD (see Sect. A.4 in the online appendix for the full robustness analysis.).

### Substantive Effect Sizes

**Fig. 2:**
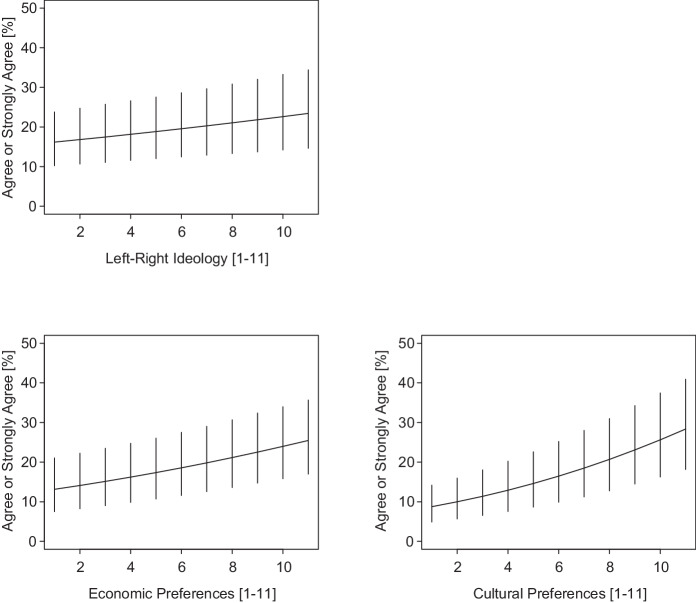
Political preferences and subjective freedom of speech. Averaged predicted probabilities with simulated 95% confidence intervals based on ordered logit model results for M15 in Table 2

Figure 2 presents how the predicted probability of feeling unfree to speak changes with political left–right ideology and economic and cultural policy preferences (along with simulated 95% confidence intervals [CIs]). Moving across the whole range of the left–right ideological scale amounts to a change from 16% (95% CI: 10%, 23%) for the far-left, to 20% (95% CI: 0.13%, 0.28%) for those in the center^17^, to 23% (95% CI: 15%, 34%) for the far right. The effect is somewhat stronger for economic policy preferences, where those on the left “more social services and higher taxes” end of the scale have only a predicted probability of 13% (95% CI: 9%, 19%) of feeling unfree to speak, those in the center have a probability of 19% (95% CI: 12%, 27%), and those on the right “lower taxes and fewer social services” end have a probability of 25% (95% CI: 16%, 36%).

But the strongest effect on subjective freedom of speech is for cultural policy preferences. Only 9% (95% CI: 5%, 14%) of the individuals in the absolute pro-immigration camp (i.e., “facilitate immigration for foreigners”) are predicted to say they cannot speak freely in public. For those with moderate views on immigration policy, 16% (95% CI: 10%, 25%) say they cannot speak freely, and for those in the absolute anti-immigration camp (i.e., “restrict immigration for foreigners”), this predicted probability rises to 28% (95% CI: 19%, 40%). This finding is also remarkable because only 5% of the population belong to the absolute pro-immigration camp, whereas the absolute anti-immigration stance is actually the second largest category, with 13% of the population (the modal middle category amounts to 14%).

**Fig. 3:**
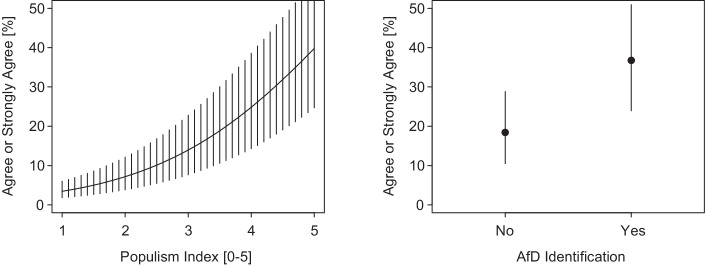
Populism, identification with the Alternative for Germany party, and subjective freedom of speech. Averaged predicted probabilities with simulated 95% confidence intervals based on ordered logit model results for M16 in Table 2

Figure 3 shows the substantive effect sizes for the remaining two robust explanations of subjective freedom of speech, namely populism and party identification with the AfD. The effect of the populism index is massive and ranges from a predicted probability of only 3% (95% CI: 2%, 6%) for those low in populism (scale point of 1) to a whopping 40% (95% CI: 24%, 57%) for those high in populism (scale point of 5). Of course, the modal category on the populism index is a scale point of 3. But even just moving from the middle category to the next scale point still amounts to a shift in a subjective lack of free speech from 14% (95% CI: 8%, 23%) to 25% (95% CI: 14%, 28%), an effect size comparable to moving across the whole range of the cultural policy preference item. The substantive effect for AfD identification is equally impressive. With a predicted probability of 37% (95% CI: 24%, 51%), AfD supporters are twice as likely as all other party supporters (18% [95% CI: 10%, 29%]) to say they are not allowed to express their opinions. We report additional exploratory analyses on the role of political preferences, populism, party identification, social media use, and personality traits in section A.5 of the online appendix.

## Discussion

One in five people in Germany state that they are no longer allowed to express their opinions freely in public. While this figure is lower than in the Allensbach survey cited in the introduction (Petersen 2021), it is still a remarkable finding for a liberal democracy that rests on the idea that all citizens should be able to voice their preferences freely. For instance, in his seminal work on the McCarthy era, Stouffer (1955) found that only 13% of the U.S. population did not feel free to speak their minds. To explain this sentiment, we preregistered and tested a wide range of hypotheses. Our results, which we summarize in Table 3, reveal that three explanatory factors are significantly, consistently, and substantively related to subjective free speech in Germany: political preferences, populist attitudes, and identification with the AfD.

**Table 3:** Summary of significant explanations for subjective freedom of speech

Explanatory variables	Models						
	Single	Group	Full	OLS	Logit	East	West
*Social class*							
Subjective social class	✔	✔	✔	✔	–	–	✔
*Political involvement*							
Political interest	✔	–	–	✔	✔	–	–
Political knowledge	✔	✔	✔	–	–	–	–
Political efficacy	✔	✔	✔	–	–	–	✔
*Political preferences*							
Left–right ideology	✔	✔	✔	✔	✔	✔	–
Economic preferences	✔	✔	✔	✔	✔	✔	✔
Cultural preferences	✔	✔	✔	✔	✔	✔	✔
Left–right ideology squared	–	–	–	–	–	–	–
Economic preferences squared	–	–	–	–	–	–	–
Cultural preferences squared	✔	✔	–	✔	–	–	–
*Personality*							
Openness to experience	✔	–	–	–	–	–	–
Neuroticism	–	–	✔	–	–	–	✔
*Social media*							
Frequency of internet use	✔	–	–	–	–	–	–
Social media platforms	–	–	–	–	–	–	–
Political participation online	✔	–	–	✔	✔	–	–
*Social and political polarization*							
Social network diversity	✔	✔	–	✔	–	–	–
*Populism*							
Populism index	✔	✔	✔	✔	✔	✔	✔
Alternative for Germany identification	✔	✔	✔	✔	✔	✔	✔

Given the polarized public discussion on free speech, it is instructive to ask how our results speak to this debate and which “side” finds more support from the empirical evidence. Is free speech in Germany stifled by a proliferation of “politically correct” speech norms and shut down by so-called cancel culture that simply cannot tolerate viewpoints it considers morally or politically beyond the pale? Or is the supposed loss of free speech merely the lament of dominant societal groups who cling on to their cultural privileges, if not a mere myth and strategic narrative spun by far-right populists who want to spread their discriminatory views? Tempting as it may be to forcefully argue for one or the other perspective, both our “adversarial collaboration” (which unites authors from both sides) and our empirical findings lead us to a more balanced assessment.

On one side, yes, it is true that the freedom to express one’s opinions is subjectively more costly for those whose political preferences do not align with or are in direct opposition to viewpoints of the cultural left. While this holds for a general conservative or right-wing ideological self-placement and libertarian economic policy preferences, it is most visible when it comes to cultural policy preferences that are critical of immigration, government measures for gender equality, climate change, and further European Union integration (see Fig. A1 in the appendix). This pattern is not simply a matter of extreme vs. moderate views or minority vs. majority opinion, either. The effects of preferences are linear, and some opinions that are considered taboo or “politically incorrect” are actually widely shared in German society (e.g., a preference for restrictive immigration policy). That views of the cultural left are less costly to express is also reflected in our explorative result for partisan identification: No one in Germany feels freer to voice their opinion in public than supporters of the Green party (see Fig. A3 in the appendix).

But of course, our data cannot judge whether these subjective perceptions are in fact accurate descriptions of the current cultural climate, and the data remain ignorant of the social sanctions that people might realistically expect. In fact, if we follow Guriev and Treisman (2019) in the assumption that more highly educated and well-informed citizens should have a more realistic grasp of the true state of free speech, there is reason to be sceptical. According to our results, higher levels of education, better political knowledge, and greater political efficacy are all related to more subjective freedom of speech—regardless of political, economic, and cultural preferences. This suggests that professing a lack of free expression is perhaps better understood as an expression of grievance than an accurate description of objective free speech. Also note that this finding differs from Gibson and Sutherland (2020), who find that in the United States it is the highly educated who report a lack of subjective free speech.

On the other side, it is also true that populism is probably the strongest and most reliable predictor of a subjective lack of free speech in current-day Germany, both in the east and the west. The two variables are so strongly related that the narrative of a loss or lack of the right to free speech must be viewed as a constitutive element of populist ideology and/or a communication strategy by populist actors. The latter argument is further supported by the finding that identification with the AfD is a very strong and robust determinant of a perceived lack of free speech. Indeed, those who identify with the populist AfD are markedly more likely to say they cannot express their opinions freely than either supporters of the liberal Free Democratic Party or supporters of the center-right Christian Democratic Union/Christian Social Union (see Fig. A3 in the appendix). However, we should also note that the cross-sectional and observational nature of our data cannot credibly establish that the causal path indeed runs from AfD identification to supporters’ subjective sense of free speech, as a model of partisan cues would suggest. In fact, if a lack of subjective free speech is an expression of political grievance, the reverse is just as plausible: Those whose voices are not heard and represented by established politics turn to populist political options instead.

In any case, it is certainly inaccurate to portray a subjective lack of free speech as the mere complaint of socially dominant groups that fear for their cultural privileges. Quite to the contrary, it is precisely those with lower social class, those with migrant backgrounds, and former East Germans who are the most likely to say they are not allowed to express their opinions publicly, regardless of their political preferences. In the absence of more detailed data on actual experiences with social sanctions, these results lend additional support to the grievance interpretation of subjective free speech. It would be prudent to take seriously the grievance that “people like them” are not free to speak, along with its democratic implications, instead of dismissing them as inaccurate or even delusional.

Of course, given the lively public debate about free speech and its limits, it is also instructive to point to hypothesized factors that do not impact citizens’ subjective sense of free speech in our analyses. First, we find little evidence for the hypothesis that subjective free speech is a matter of psychological disposition or personality. Neither the personality trait of openness nor the neuroticism dimension is consistently related to the perceived freedom to speak one’s mind. This is an important result because it is sometimes assumed that those who complain about a lack of free speech are just too sensitive, lack resilience, and simply cannot handle contradiction. Our findings suggest that this psychological characterization is inaccurate.^18^

Second, and importantly, our empirical results also provide little support for the hypothesis that links new online communication technology and social media platforms to citizens’ sense of free speech or a lack thereof. Neither frequent internet use, nor the number of social media platforms, nor active online political participation is robustly related to subjective freedom of speech, once other social and political explanations are accounted for. This finding is especially noteworthy because social media is often not only the main suspect in public debates on free speech but also the subject of new regulatory measures and content-based speech restrictions. Our results suggest that social media is probably not perceived as a distinct arena but is viewed as a natural extension of the public sphere. However, we should also note that we found pronounced differences across social media platforms in additional exploratory analyses (see Fig. A4 and discussion in the appendix). One possible explanation for diverging platform effects lies in varying levels of homophily in users’ communication networks and different degrees of “echo chamber effects” (Sunstein 2017; Cinelli et al. 2021).

But third, and directly related to this last point, we actually find little consistent evidence that less polarized and more diverse social networks are particularly conducive to subjective freedom of speech. This casts some doubt on the notion that opportunities for “hearing the other side” are a natural and logical solution to the perceived free speech crisis. To be sure, network diversity is only one mechanism that connects political polarization to citizens’ subjective sense of free speech. With the data at hand, we were not able to take a deeper look at the political identification processes underlying political polarization and how they relate to the subjective costs of speaking one’s mind in public. It is this and the other questions we raised in the discussion that we would like to scrutinize in future work.

## Supplementary Information

Online Appendix

## Footnotes

1 Since unification in 1990, Germany has a near-perfect score (0.97 out of 1) on the *Freedom of Discussion* indicator of the V‑Dem project. On the *Freedom of Expression Index*, which considers state censorship efforts, harassment of journalists, media self-censorship, freedom of discussion for men and women, and freedom of academic and cultural expression, Germany scored 0.96 in 2020—only a miniscule decline compared to the score of 0.98 for the period 1990–2014 and 0.97 for the period 2015–2019. Alternative indices, such as those provided by Freedom House, the Polity5 Project, and Reporters Without Borders, produce similar results.

2 See Gibson and Sutherland (2020) for comparable trends in the United States: In 1973 some 80% reported they “feel as free to speak their mind as they used to,” but in 2019 only 60% of the population perceived subjective freedom of speech.

3 Of course, this normative prior on the democratic desirability of subjective freedom of speech does not imply that all speech is equally desirable or in fact deserves to be heard and protected. But for the purposes of the present study, we remain agnostic about the intricacies of the complex normative trade-offs the topic involves.

4 The preregistration can be found on OSF under https://osf.io/8fkh9 and full replication code under https://osf.io/s82jv/.

5 We answered the same political preference items that the GLES asks its respondents (see Table A.2.2 in the online appendix for exact wording and answer scales). While one author scores a 2 on the ideological left–right scale, a 2 on the cultural dimension, and an 11 on the economic dimension, the other author scores an 8 on the left–right scale, a 6 on the cultural dimension, and a 3 on the economic dimension.

6 For a similar logic of adversarial collaboration on free expression, but in the more narrowly defined university context, see Revers and Traunmüller (2020), Traunmüller and Revers (2021), Villa et al. (2021), and, most notably, Wuttke et al. (2021).

7 Self-censorship is a *behavioral consequence* of, and therefore conceptually distinct from, a lack of subjective freedom of speech. Individuals who do not feel free to express themselves will instead try to hide their true preferences (Kuran 1997) or will avoid speaking about politics with their social contacts and fellow citizens altogether (Hayes et al. 2006). But self-censorship is not *deterministically* related to subjective freedom of speech. One may feel unfree and still (or in spite) have the courage to speak out. Conversely, one may feel perfectly free to speak but still choose to self-censor. Alternative motives for self-censorship may include the desire not to hurt someone else’s feelings, to further a specific cause, or to protect an individual, group, or organization (Bar-Tal 2017). In addition, different motives may matter for private and public speech.

8 We should note that Gibson relied on a somewhat broader conceptualization of subjective freedom that includes both perceptions of governmental censorship and actual self-censorship on political matters. This and the fact that his analysis refers to the U.S. population in the 1990s suggests that our “replication” is meant in the wider sense of the term. Yet we expect that his arguments are general enough to also hold for the case of present-day Germany.

9 At the same time, we want to stress that the reverse may also be plausible. As Ong (2021) argues, individuals with higher socioeconomic status may actually perceive the penalty of sanction, C_sanction_, to be greater than those in a lower social class do. The reason is that because they have higher income, higher anticipated future income, and greater reputation to lose, they risk much more if they speak freely and get sanctioned for it. Therefore, as long as perceived B_express_, C_express_, and p_sanction_ are held constant, those higher in social class should be more likely to think that they cannot speak freely.

10 We argue that “openness to experience” corresponds closely to “closed-mindedness” (when reverse-coded, of course) because it describes the complexity of an individual’s mental life (John et al. 2008). Furthermore, while strictly speaking they are distinct constructs, we proxy a lack of “self-esteem” with “neuroticism,” which distinguishes individuals by their confidence, emotional stability, and ego strength as well as their predisposition for negative emotionality (John et al. 2008).

11 Because of word limit constraints, the variable descriptions and paragraphs from the sections on *data exclusion and missing data *and *sampling weights *are shortened. Their original, preregistered wording is in the online appendix (A.1).

12 The Allensbach item (Petersen 2021) requires respondents to trade off free expression with caution: “Do you feel that in Germany today you can freely express your political opinion, or is it better to be cautious?” Arguably, these two concepts do not form opposing poles on the same underlying continuum, because one can feel free to speak and still think that it is a good idea to be cautious when doing so (Schneider 2021).

13 Because of word limit constraints, the paragraphs on *effect size, statistical power, *and *assumption violation/model nonconvergence *have been moved to the online appendix in their original, preregistered wording (A.1).

14 We use the correction $q=p\times m/i$, where *m* is the number of tests and *i* is the rank of the ordered *p*-values. As critical threshold for these so-called *q*-values we set 0.10, which corresponds to a false discovery rate of 10%.

15 While the preregistered criterion for our hypotheses tests is M16 and the respective robustness checks, we afterward chose to also rerun model M15 with ordinal logistic regression and logistic-regression specifications (see Table A.3.5 in the online appendix) and separate for eastern and western Germany (Table A.3.6 in the online appendix).

16 We also ran both measures separately (M11A, M11B), producing equal results (populism index: estimate 1.18, standard error 0.04, q‑value 0.00*; AfD identification: estimate 2.41, standard error 0.13, q‑value 0.00*). These two models were not preregistered but are included in the syntax.

17 This corresponds to the modal scale point of 6 on the left–right scale.

18 We also explored the remaining three Big Five personality dimensions of conscientiousness, agreeableness, and extroversion and report the results in Fig. A5 in the appendix.
