# Supplementary material for: Subjective Freedom of Speech: Why Do Citizens Think They Cannot Speak Freely?
Source: Polit Vierteljahresschr. 2022 Aug 11;64(1):155–81. doi: 10.1007/s11615-022-00414-6 (PMC9368691; doi:10.1007/s11615-022-00414-6)
Supplement: Supplementary file 1 — Online Appendix [file 11615_2022_414_MOESM1_ESM.docx]

**Online Appendix**

**A.1 Original Wording of Pre-Registered Data, Methods & Analysis Plan**

*a) Data*

For our analyses we rely on the *GLES 2021 Cross-Section Pre-Election Surve*y (GLES 2022) which was fielded in between the 26^th^ of August and the 25^th^ of September 2021. 5116 respondents were sampled from German citizens aged 16 years or older and living in private households using a multi-stage register sample. Data was collected using a mixed-mode design including both computer-assisted web-based interviews (CAWI) and paper-and-pencil interviews (PAPI). Interview participation was incentivized with 5 Euros paid unconditionally before the interview.

*b) Variables*

*Subjective Freedom of Speech*

The variable of main interest to our study is a newly designed survey item of subjective freedom of speech: “People like me are no longer allowed to express their opinions freely in public.” It is part of an item battery on feelings of marginalization including a total of four items. Respondents are asked to rate their agreement on a 5-point scale which we reverse such that higher scores indicate higher levels of agreement. Substantively, higher levels of agreement indicate a greater lack of subjective freedom of speech. This new item circumvents some of the problems and ambiguities surrounding the validity of previous question wordings.^^[[1]](#footnote-1)^^

In addition, the *GLES 2021 Cross-Section Pre-Election Surve*y provides a wide range of relevant covariates that allow us to evaluate the question why citizens think they cannot speak freely. In general, we treat most ordered categorical variables or count variables as quasi-continuous and assume linear effects. Composite scales combining several items are constructed using simple unweighted averages. Here, we only briefly discuss the variables used in our analyses. We refer to tables A.2.1-A.2.4 for questionnaire variable numbers, exact question wordings, answer scales as well as more detailed coding decisions. Descriptive statistics including number of observations, missing values, means, standard deviations, minimum and maximum variable values are included in table A.3.0.

*Explanatory Variables*

To explain subjective freedom of speech, we operationalize *social class* using a subjective measure asking respondents to identify which out of six social classes they feel they belong to. For simplicity, we enter this variable as quasi-continuous measure and assume a linear effect. *Political involvement* is measured using a total of three variables: respondents’ general political interest (reverse-coded and treated as quasi-continuous with linear effect), political knowledge (a binary answer to a simple question on the German electoral system) and a survey item on political efficacy (reverse-coded, assumed quasi-continuous with linear effect). To tap into respondents’ *political preferences*, we rely on the standard 11-point ideological left-right scale. We enter this variable both linearly and with an additional quadratic term. For more nuance in measuring political preferences, we also distinguish between the economic and the cultural dimension of the political preference space. The economic dimension is measured using a reverse-coded item on the trade-off between social services and taxes, the cultural dimension is measured using an item on immigration preferences. Both variables are measured on 11-point scales and entered as linear and quadratic term.^^[[2]](#footnote-2)^^

To measure respondents’ *personality* we rely on a Big Five short scale where each of the personality dimensions is captured using two items. Openness to experience is measured by asking respondents whether they are imaginative and whether they have artistic interests. Neuroticism is measured with two items eliciting respondents’ ability to handle stress and easily getting nervous. We do not conduct a factor analysis to verify the factor structure. Instead, we construct simple mean scores for each personality dimension (reverse coding one of the items in each dimension).^^[[3]](#footnote-3)^^

We test our *social media use* hypothesis using three items. First, respondents are asked on how many days a week they use the internet. Second, they can indicate which social media platforms they use (out of a list of fourteen different platforms, including Facebook, Twitter, Instagram, etc.). We recode these responses into a platform count-variable ranging from 0 to 14, thus assuming equal importance for all platforms^[[4]](#footnote-4)^. Third, respondents are asked whether they partook in varying forms of political participation online with the first three sub-items directly relating to social media posting, sharing and liking of political content, respectively. We again recode responses to those three questions into a count-variable ranging from 0 to 3, assuming equal importance for all three forms of signaling one’s opinion. Subsequently, all three variables are treated as quasi-continuous, again assuming linear effects.

*Political and social polarization* is operationalized by the degree of opinion diversity in respondents’ discussion network. Using an ego-centered network generator, respondents are asked how often they disagree with the first and second person they name as their political discussion partners. The answers for the two network alteri are averaged. Respondents with no political discussion partner are assigned the lowest score (i.e. ‘no opinion diversity in social network’). Respondents’ affinity to *populism* is captured using a scale consisting of a total of six items tapping into several relevant aspects of populism such as cynicism toward political elites and preferences for popular sovereignty. For scale construction we use a simple unweighted average score, which we enter into the model equations in linear form.

In order to measure identification with the AfD we rely on an item surveying party identification regardless of recent voting decision. From this, we code a binary variable indicating whether the respondent identifies with the AfD or not.

*Control Variables*

For the models explaining subjective freedom of speech, we control for several key socio-demographics which may be related to both, subjective freedom of speech and the hypothesized explanatory variables (and which have also been used for constructing survey weights, see below). Specifically, we include respondents’ gender, age, and education along with a simple dummy for respondents from Eastern parts of Germany. In addition, we include respondents’ migration background (i.e. whether they themselves or one of their parents were born abroad) and whether they live in a rural or urban environment.

*c) Data Exclusion and Missing Data*

In the previous *GLES 2017 Cross-Section Pre-Election Survey* no less than 36 percent of all respondents had at least one ‘don’t know’ or ‘no answer’ among the set of covariates that we use in our analyses. We expected similar rates of item non-response in the 2021 survey. Thus, using list-wise deletion in models with many covariates was likely to result in a considerable loss of information. We therefore treat ‘don’t knows’ as missing values (unless explicitly stated otherwise in the coding instructions, see tables A.2.1-A.2.4) and employ multiple imputation using chained equations (Van Buuren 2007). Specifically, we conduct M=5 imputations using the subset of explanatory and control variables described in the previous section (excluding the subjective freedom of speech item), run our models on all five imputed data sets and present the combined results (Little & Rubin 1987).

*d) Sampling Weights*

The *GLES 2021 Cross-Section Pre-Election Surve*y provides both a design weight and adjustment weights. Whereas the design weight corrects for oversampling in East Germany, the adjustment weights correct for deviations from the population distributions of gender, age, education, BIK regions and East-West. Except for describing the answer distribution in our outcome variable which we report using the adjustment weights (see Figure 1), we do not explicitly use survey weighting in our analyses. Instead, we include all variables used for the weighting as control variables in our regression models to adjust for any sample imbalances (see Gelman 2007).^^[[5]](#footnote-5)^^

**Analysis Plan**

*a) Statistical Models*

We rely on ordered logit models as our default modeling choice (see more on robustness checks further below). We start out with a model that only includes the control variables to get an impression on the socio-demographic distribution of the perception that one cannot speak freely (see table A.3.1). In a next step, we estimate models for each hypothesis *separately*, each time including the respective explanatory variable(s) along with the controls (M1-M11, see table A.3.2). The final model specifications are joint models including either subsets of (M12-14, table A.3.3), or all (M15-16, table 1) explanatory variables along with the controls.

*b) Effect Size*

While we had no specific prior expectations about the relative or absolute effect sizes of the many hypothesized explanatory factors, we provide meaningful quantities of interest along with their inferential uncertainties in the presentation of our results (King et al. 2000). Importantly, for all models using subjective freedom of speech as ordered categorical outcome, we provide and visualize predicted probabilities for ‘agreeing’ or ‘strongly agreeing’ using and averaging over the actual observed individual covariate values (Hanmer & Kalkan 2013). Inferential uncertainties in terms of 95 percent confidence intervals are obtained using statistical simulation based on draws from a multivariate normal distribution.

*c) Statistical Power*

Since our analysis plan refers to survey data where the planned sample size has already been determined, we were not in the position to adapt the data collection based on an a priori power calculation. While in principle an ex-post power calculation could be of interest to determine minimally detectable effects or whether any failed hypothesis tests are really due to low power, our reasoning is as follows. Given the planned large number of respondents for the survey (N=5116), we are likely to only miss very small effects – effects with only little practical relevance. In short, we do not conduct any ex-post power analyses.

*d) Inference Criteria*

For statistical inference, we rely on classical frequentist p-values. However, given the multitude of hypotheses we plan to test, we heed the multiple comparisons problem and correct these p-values to guard against false discoveries. Specifically, we rely on the Benjamini and Hochberg (1995) procedure to control the false discovery rate (FDR), i.e. the expected proportion of false discoveries amongst all significant hypotheses.^^[[6]](#footnote-6)^^ In defining the first ‘family’ of tests, we count all tests for explanatory variables (not the control variables) in the model specifications M1-M16. Each set of robustness tests is treated as its own ‘family’ of tests. We judge hypotheses as having survived if a) at least one of its respective indicators reaches statistical significance in b) the full model specification M16 and c) remains robust under the two alternative specifications suggested as robustness tests (see below).

*e) Reliability and Robustness Testing*

In our robustness tests we first focus on our key variable, subjective freedom of speech, and gauge how our inferences change when we alter its assumed measurement scale. While by default we model the subjective freedom of speech survey item as an ordered categorical outcome, we check the robustness of our findings when treating it a) as quasi-continuous in a linear regression model and b) as a binary variable in a logistic regression model (see table A.3.4). In the latter case, we join the ‘agree’ and ‘strongly agree’ categories and contrast them with the remaining three categories. For these robustness tests we only re-run model specification M16. Given the high number of hypotheses, we apply a very strict robustness criterion and only report a given result as ‘robust’ when it reaches statistical significance in all three alternative specifications.^^[[7]](#footnote-7)^^ For a second, more substantively motivated, robustness test we run separate specifications of M16 for East and West (omitting the East-West dummy of course, see table A.3.5).^[[8]](#footnote-8)^

*f) Assumption Violation/Model Non-Convergence*

Since our analysis relies on a single cross-sectional data set of large size, the most serious violations we expected to encounter in our statistical models are wrong functional form assumptions for our explanatory variables. We were only able to detect them ex-post. While we document any deviations from the linearity assumption (see table A.3.8), we report these findings as purely explorative and do not change any hypotheses based on these insights. Any computational convergence issues are documented. Since we proposed and pre-registered several alternative model specifications as robustness checks, the reported results are then solely based on the models without convergence issues. In the cases of perfect collinearity or perfect separation we a priori decided to turn to Bayesian specifications with weakly informative priors as a remedy. However, no such problems occurred during the analysis.

**A.2 Variables and their coding**

**A.2.1 Outcome Variable**

| **Left behind: subjective, no Freedom of Expression (Q46d)**  To what extent do you agree with the following statements or not?  (D) People like me are no longer allowed to freely express their opinions in public.   1. Strongly agree 2. Agree 3. Neither agree nor disagree 4. Disagree 5. Strongly disagree   (-99) No answer  *Coding: Values treated as ordinal scale, reverse-coded* |
| --- |

**A.2.2 Explanatory Variables**

| H_1_ | **Class Affiliation: subjective, personal (D38)**  People talk a lot about social classes these days. Which of these classes do you consider yourself a part of?   1. Lower class 2. Working class 3. Lower middle class 4. Middle Class 5. Upper middle class 6. Upper class   (-99) No answer  *Coding: Treated as quasi-continuous, linearity assumed* |
| --- | --- |
| H_2_ | **Political Interest (Q1)**  Quite generally, how interested are you in politics?   1. Very interested 2. Somewhat interested 3. In between 4. Not very interested 5. Not at all interested   (-99) No answer  *Coding: Treated as quasi-continuous, linearity assumed , reverse-coded* |
|  | **Political Knowledge: First/Second Vote (Q5)**  In the federal elections you have two votes, the first vote and the second vote. What do you think: Which vote decides how many seats each party will have in parliament?   1. The first vote 2. The second vote 3. Both are equally important   (-98) Don’t know  (-99) No answer  *Coding: Binary; correct answer (2) = 1, wrong answers and (-98) = 0* |
|  | **Attitudes: Politics in General, political Issues difficult to Understand (Q50a)**  Now some general statements about politics. Please indicate how much you agree or disagree with each of these statements.   1. I often find political issues difficult to understand. 2. Strongly disagree 3. Disagree 4. Neither agree nor disagree 5. Agree 6. Strongly agree   (-99) No answer  *Coding: Treated as quasi-continuous, linearity assumed, reverse-coded* |
| H_3_  _&_  H_4_ | **Left-Right Assessment: Ego (Q37)**  Where would you place yourself on this scale?   1. 1 Left 2. 2 3. …   (10) 10  (11) 11 Right  (-97) Not applicable  (-98) Don’t know  (-99) No answer  **Position Issue: Taxes vs. Social Services, Ego (Q40)**  And what position do you take on taxes and social services?   1. 1 Lower taxes and fewer social services 2. 2 3. …   (10) 10  (11) 11 More social services and higher taxes  (-99) No answer  **Position Issue: Immigration, Ego (Q43)**  And what position do you take on immigration for foreigners?   1. 1 Facilitate immigration for foreigners 2. 2 3. …   (10) 10  (11) 11 Restrict immigration for foreigners  (-99) No answer  *Coding for (H_3_): for each variable linearity is assumed, Q40 is reverse-coded*  *Coding for (H_4_): the squared term for each variable from (H_3_) is added* |
| H_5_ | **Psychological Concepts: Big Five (Q80 – e & j)**  Now to something completely different: To what extent do the following statements apply to you?  (e) I have an active imagination and am imaginative.  (j) I have few artistic interests.   1. Does not apply at all 2. Rather does not apply 3. Neither applies nor does not apply 4. Rather applies 5. Applies completely   (-99) No answer  *Coding: Treated as quasi-continuous, linearity and equal importance of both items assumed; (j) is reverse-coded and mean-value is taken* |
| H_6_ | **Psychological Concepts: Big Five (Q80 – d & i)**  Now to something completely different: To what extent do the following statements apply to you?  (d) I am relaxed and can handle stress well.  (i) I easily get nervous and uneasy.   1. Does not apply at all 2. Rather does not apply 3. Neither applies nor does not apply 4. Rather applies 5. Applies completely   (-99) No answer  *Coding: Treated as quasi-continuous, linearity and equal importance of both items assumed; (d) is reverse-coded and mean-value is taken* |
| H_7_ | **Internet: Use, on Average (Q68)**  On how many days per week, on average, do you use the Internet?   1. Less than 1 day per week 2. 1 day 3. …   (7) 7 days  (8) Never use the internet  (9) No internet access  (-99) No answer  *Coding: Treated as quasi-continuous and linearity assumed; (8) and (9) are recoded as the lowest value; below “less than 1 day per week”*  **Social Media: Usage (Q70)**  Which social media platform do you use occasionally or regularly?   1. WhatsApp 2. Facebook 3. Instagram 4. Youtube 5. Twitter 6. Snapchat 7. TikTok 8. Telegram 9. Signal 10. Threema 11. XING 12. LinkedIn   (M) Discord  (N) Others, namely  (X) I don’t use social media   1. Mentioned 2. Not mentioned   (-97) Not applicable  (-99) No answer  *Coding: Equal importance of all items (A-N) assumed; sum of indicated items is taken; if X is indicated overall value is coded as 0*  **Participation: Online (Q72, A-C)**  Let’s look now at political participation on the Internet. Here are some ways of being politically active online. Please say whether you have used any of these ways within the last twelve months. Please indicate if you don’t use the Internet. Have you…?   1. Expressed your own political views in posts on social media, in tweets or using a mailing list 2. Forwarded or shared other people’s political views on social media 3. Clicked “Like” for political views expressed in social media 4. Yes 5. No   (-73) I never use the internet  (0) I have not been politically active in any of these ways  (-99) No answer  *Coding: Equal importance of all items (A-C ) assumed; items with value (1) are summed up; if (-73) or (0) is chosen, overall value is set to 0, linearity assumed* |
| H_8_ | **Personal Networks: Person 1, Differences of opinion (Q93)**  **Personal Networks: Person 2, Differences of opinion (Q97)**  When you talk to this person about political questions, how often would you say that you are of different opinions?   1. Often 2. Sometimes 3. Rarely 4. Never   (-97) Not applicable  (-99) No answer  *Coding: Treated as quasi-continuous and linearity assumed;*  *- Original scales are reverse-coded*  *- Respondents talking to no person receive (0)*  *- Respondents talking to one person receive that indicated value*  *- Respondents talking to two persons receive mean of indicated values* |
| H_9_ | **Attitudes: Populism (Q51)**  Please say how much you agree or disagree with each of these statements.   1. What people call compromise in politics is really just selling out on one’s principles. 2. The people, and not politicians, should make our most important policy decisions. 3. The politicians in the German Bundestag need to follow the will of the people. 4. The political differences between the elite and the people are larger than the differences among the people 5. I would rather be represented by a citizen than by a specialized politician 6. Politicians talk too much and take too little action. 7. Strongly disagree 8. Disagree 9. Neither agree nor disagree 10. Agree 11. Strongly agree   (-99) No answer  *Coding: Treated as quasi-continuous, linearity and equal importance of items assumed; mean-value over all items (A-F) is taken* |
| H_10_ | **Party Identification (Q75)**  Now, let's look at the political parties. In Germany, many people lean towards a particular party for a long time, although they may occasionally vote for a different party. How about you, do you in general lean towards a particular party? If so, which one?   1. CDU/CSU 2. CDU 3. CSU 4. SPD 5. AfD 6. FDP 7. DIE LINKE 8. GRÜNE   (801) Other party, namely _______________  (808) No party  (-99) No answer  Coding: *Transformed into binary variable indicating existence of identification with AfD* |

**A.2.3 Variables for exploratory analyses**

| Issues  &  Attitudes | **Attitudes: Issue Battery (Q27)**  There are various opinions on different political issues. What do you think of the following statements?   1. Immigrants should be obliged to assimilate into the German culture. 2. There should be a statutory quota of women for the supervisory boards of large companies. 3. The state should stay out of the economy. 4. The government should take measures to reduce differences in income levels. 5. Citizens should be able to induce a binding referendum at federal level. 6. European unification should be pushed further. 7. Governmental measures for gender equality in society already go too far. 8. To combat climate change, taxes on fossil fuels such as oil, gas and coal should be increased. 9. The government should implement a nationwide rent cap. 10. In times of a pandemic like Corona, there should be a universal vaccination requirement. 11. In emergency situations, it is justified for the state to restrict citizens' civil liberties. 12. Strongly agree 13. Agree 14. Neither agree nor disagree 15. Disagree 16. Strongly disagree   (-99) No answer |
| --- | --- |
| Big Five | **Psychological Concepts: Big Five (Q80a-c, f-h)**  Now to something completely different. To what extent do the following statements apply to you?   1. I tend to be somewhat shy and reserved. 2. I easily trust other people and see the good in others. 3. I perform tasks very thoroughly.   (F) I am outgoing and sociable.  (G) I tend to find fault with others.  (H) I tend to be lazy.   1. Does not apply at all 2. Rather does not apply 3. Neither applies nor does not apply 4. Rather applies 5. Applies completely   (-99) No answer  *Coding: Treated as quasi-continuous, linearity and equal importance of items assumed*  *- mean value of (f) and reverse-coded (a) creates variable “extraversion”*  *- mean value of (c) and reverse-coded (h) creates variable “conscientiousness”*  *- mean value of (b) and reverse-coded (g) creates variable “agreeableness”* |

**A.2.4 Control Variables**

| **Gender/Sex (D1)**  First, two questions about yourself. Please indicate your gender.   1. Male 2. Female 3. Diverse   (-99) Don’t want to answer question  *Coding: As GLES randomly assigns “diverse” respondents into categories “male” and “female” for data protection reasons, sex is included as a binary variable, (-99) is treated as missing.* |
| --- |
| **Date of Birth: Year, Month, Day (D2)**  Please indicate on which day, month, and year you were born.  Day:   1. 1 2. 2 3. …   (31) 31  Month:   1. January 2. …   (12) December  Year:  (1910) 1910  (1911) …  (2005) 2005  (-99) No answer  *Coding: Age is calculated by first subtracting the respondent’s birth-year from the survey-year (2021) and then grouping respondents as follows:*   1. *16-29 years* 2. *30-44 years* 3. *45-59 years* 4. *60 years and older* |
| **Education: School (D7)**  What's your highest level of general education?   1. Finished school without school leaving certificate 2. Lowest formal qualification of Germany’s tripartite secondary school system, after 8 or 9 years of schooling (“Hauptschulabschluss”, “Volksschulabschluss”) 3. Intermediary secondary qualification, after 10 years of schooling (“Mittlere Reife”, “Realschulabschluss” resp. “Polytechnische Oberschule mit Abschluss 10. Klasse”) 4. Certificate fulfilling entrance requirements to study at a polytechnical college (“Fachhochschulreife (Abschluss einer Fachoberschule etc.)”) 5. Higher qualification, entitling holders to study at a university (“Abitur” resp. “Erweiterte Oberschule mit Abschluss 12. Klasse (Hochschulreife)”)   (9) Still at school  (6) Other school leaving certificate, please enter:_______  (-99) No answer  *Coding: Education is coded along the lines of the tripartite secondary school system*  *- (1), (2) & (9) combine to form category “low”*  *- (3) forms category “medium”*  *- (4) & (5) combine to form category “high”*  *- (6) will be treated as missing, since the coding of open answers will not be available prior to the release of the first data set version* |
| **Residence: urban-rural (Wum6)**  Which of the following categories best describes where you live?   1. Large city 2. Outskirts or suburbs of a large city 3. Medium or small town 4. Rural village 5. Single homestead or detached house in the countryside   (-99) No answer  *Coding: Recoded into the following ordinal scale:*  *- (4) & (5) are subsumed in category (1) “rural”*  *- (3) becomes category (2) “town”*  *- (1) & (2) are subsumed in category (3) “city”* |
| **Eastern and Western Germany**  *Coding: Here, we use the East/West dummy-variable automatically generated by GLES and let (1) denote Eastern and (0) Western Germany* |
| **Citizenship: German, since Birth (D42)**  Have you been a German citizen since birth?   1. Yes 2. No   (-99) No answer  **Country of Birth: Germany, Parents (D49)**  Were both your parents born on current German territory?   1. Yes 2. No, mother was not born on current German territory 3. No, father was not born on current German territory 4. No, mother and father were not born on current German territory   (-98) Don’t know  (-99) No answer  *Coding: Migration Background is coded binary, denoting whether a respondent or either of their parents was not born in Germany (1) or all were born in Germany (0)*  *(i.e., if D42 is (1) & D49 is (1) the resulting code is (0), all other combinations result in (1))* |

**A.3 Additional Tables**

Table A.3.0: Descriptive Statistics (before Imputation)

|  | N | Missing (%) | Mean | SD | Min | Max |
| --- | --- | --- | --- | --- | --- | --- |
| Subjective Freedom of Speech | 5,065 | 1.00 | 2.19 | 1.28 | 1 | 5 |
| Subjective Social Class | 4,942 | 3.40 | 3.69 | 0.99 | 1 | 6 |
| Political Interest | 5,078 | 0.74 | 3.37 | 0.85 | 1 | 5 |
| Political Knowledge | 5,065 | 1.00 | 0.56 | 0.50 | 0 | 1 |
| Political Efficacy | 5,085 | 0.61 | 3.39 | 1.00 | 1 | 5 |
| Left-Right Ideology | 4,704 | 8.05 | 5.41 | 2.00 | 1 | 11 |
| Economic Preference | 5,031 | 1.66 | 5.79 | 2.29 | 1 | 11 |
| Cultural Preference | 5,060 | 1.09 | 6.49 | 2.88 | 1 | 11 |
| Openness to Experience | 4,969 | 2.87 | 3.41 | 0.89 | 1 | 5 |
| Neuroticism | 4,979 | 2.68 | 2.64 | 0.82 | 1 | 5 |
| Frequency of internet use | 5,021 | 1.86 | 6.90 | 2.36 | 0 | 8 |
| Social media platforms | 5,062 | 1.06 | 2.70 | 2.00 | 0 | 12 |
| Online Political Participation | 4,818 | 5.82 | 0.47 | 0.82 | 0 | 3 |
| Social Network Diversity | 4,704 | 8.05 | 2.35 | 1.04 | 0 | 4 |
| Populism Index | 4,892 | 4.38 | 3.35 | 0.74 | 1 | 5 |
| Party Identification (AfD) | 4,964 | 2.97 | 0.05 | 0.21 | 0 | 1 |
| Female | 5,068 | 0.94 | 0.48 | 0.50 | 0 | 1 |
| Age | 5,030 | 1.68 | 52.24 | 18.33 | 16 | 90 |
| Education | 4,928 | 3.67 | 4.67 | 1.25 | 1 | 6 |
| East Germany | 5,116 | 0.00 | 0.33 | 0.47 | 0 | 1 |
| Rural Residence | 5,000 | 2.27 | 3.26 | 1.14 | 1 | 5 |
| Migrant Background | 4,913 | 3.97 | 0.24 | 0.43 | 0 | 1 |

Table A.3.1: Ordered Logistic Regression of Subjective Freedom of Speech on Control Variables

|  | Est. | SE |
| --- | --- | --- |
| Female | -0.01 | 0.05 |
| Age: 30-44 | 0.31 | 0.09 |
| Age: 45-59 | 0.09 | 0.09 |
| Age: 60 and over | 0.00 | 0.09 |
| Education: Medium | -0.13 | 0.08 |
| Education: High | -0.88 | 0.08 |
| Small Town | -0.14 | 0.07 |
| Large City | -0.40 | 0.07 |
| East Germany | 0.51 | 0.06 |
| Migrant Background | 0.16 | 0.06 |

Table A.3.2: Separate Results: Explanations of Subjective Freedom of Speech (Ordered Logistic Regressions)

|  | Est. | SE | q |
| --- | --- | --- | --- |
| *Social Class* |  |  |  |
| M1: Subjective Social Class | -0.24 | 0.03 | 0.00* |
| *Political Involvement* |  |  |  |
| M2: Political Interest | -0.16 | 0.03 | 0.00* |
| M3: Political Knowledge | -0.36 | 0.05 | 0.00* |
| M4: Political Efficacy | -0.22 | 0.03 | 0.00* |
| *Political Preferences* |  |  |  |
| M5: Left-Right-Ideology | 0.05 | 0.02 | 0.01* |
| M5: Economic Preferences | 0.12 | 0.01 | 0.00* |
| M5: Cultural Preferences | 0.26 | 0.01 | 0.00* |
| M6: Left-Right-Ideology | 0.02 | 0.06 | 0.86 |
| M6: Economic Preferences | 0.06 | 0.05 | 0.35 |
| M6: Cultural Preferences | 0.00 | 0.05 | 0.98 |
| M6: Left-Right-Ideology squared | 0.00 | 0.01 | 0.64 |
| M6: Economic Preferences squared | 0.00 | 0.00 | 0.35 |
| M6: Cultural Preferences squared | 0.02 | 0.00 | 0.00* |
| *Personality* |  |  |  |
| M7: Openness to Experience | -0.13 | 0.03 | 0.00* |
| M8: Neuroticism | 0.03 | 0.03 | 0.44 |
| *Social Media* |  |  |  |
| M9: Frequency of Internet Use | -0.03 | 0.01 | 0.04* |
| M9: Social Media Platforms | -0.03 | 0.02 | 0.14 |
| M9: Political Participation Online | 0.12 | 0.04 | 0.00* |
| *Social & Political Polarization* |  |  |  |
| M10: Social Network Diversity | -0.12 | 0.03 | 0.00* |
| *Populism* |  |  |  |
| M11: Populism Index | 1.08 | 0.04 | 0.00* |
| M11: AfD Identification | 1.81 | 0.13 | 0.00* |
| *Socio-Demographic Controls* |  | *yes* |  |

*Note:* * *q*-values < .1 (i.e. corrected *p*-values that allow for a false discovery rate among significant findings of maximum 10 percent).

Table A.3.3: Robustness: Explanations of Subjective Freedom of Speech

|  | | OLS |  | | Logit |  |
| --- | --- | --- | --- | --- | --- | --- |
|  | Est. | SE | q | Est. | SE | q |
| *Social Class* |  |  |  |  |  |  |
| Subjective Social Class | -0.06 | 0.02 | 0.01* | -0.09 | 0.05 | 0.17 |
| *Political Involvement* |  |  |  |  |  |  |
| Political Interest | 0.05 | 0.02 | 0.08* | 0.21 | 0.06 | 0.00* |
| Political Knowledge | -0.06 | 0.03 | 0.14 | 0.04 | 0.09 | 0.81 |
| Political Efficacy | -0.02 | 0.02 | 0.39 | 0.05 | 0.05 | 0.52 |
| *Political Preferences* |  |  |  |  |  |  |
| Left-Right-Ideology | 0.09 | 0.03 | 0.04* | 0.24 | 0.10 | 0.05* |
| Economic Preferences | 0.01 | 0.03 | 0.83 | -0.01 | 0.08 | 0.93 |
| Cultural Preferences | -0.01 | 0.03 | 0.85 | 0.02 | 0.08 | 0.83 |
| Left-Right-Ideology squared | 0.00 | 0.00 | 0.21 | -0.01 | 0.01 | 0.21 |
| Economic Preferences squared | 0.00 | 0.00 | 0.21 | 0.01 | 0.01 | 0.39 |
| Cultural Preferences squared | 0.01 | 0.00 | 0.00* | 0.01 | 0.01 | 0.13 |
| *Personality* |  |  |  |  |  |  |
| Openness to Experience | -0.02 | 0.02 | 0.52 | -0.02 | 0.05 | 0.83 |
| Neuroticism | 0.03 | 0.02 | 0.21 | 0.04 | 0.06 | 0.70 |
| *Social Media* |  |  |  |  |  |  |
| Frequency of Internet Use | 0.00 | 0.01 | 0.81 | 0.01 | 0.02 | 0.78 |
| Social Media Platforms | 0.00 | 0.01 | 0.83 | 0.03 | 0.03 | 0.52 |
| Political Participation Online | 0.05 | 0.02 | 0.04* | 0.15 | 0.06 | 0.04* |
| *Social & Political Polarization* |  |  |  |  |  |  |
| Social Network Diversity | -0.03 | 0.02 | 0.08* | -0.05 | 0.04 | 0.42 |
| *Populism* |  |  |  |  |  |  |
| Populism Index | 0.44 | 0.02 | 0.00* | 0.96 | 0.07 | 0.00* |
| AfD Identification | 0.83 | 0.08 | 0.00* | 1.19 | 0.18 | 0.00* |
| *Socio-Demographic Controls* |  | *yes* |  |  | *yes* |  |

*Note:* * *q*-values < .1 (i.e. corrected *p*-values that allow for a false discovery rate among significant findings of maximum 10 percent).

Table A.3.4: East and West Differences: Explanations of Subjective Freedom of Speech (Ordered Logistic Regressions)

|  | | East |  | | West |  |
| --- | --- | --- | --- | --- | --- | --- |
|  | Est. | SE | q | Est. | SE | q |
| *Social Class* |  |  |  |  |  |  |
| Subjective Social Class | -0.09 | 0.05 | 0.25 | -0.12 | 0.04 | 0.02* |
| *Political Involvement* |  |  |  |  |  |  |
| Political Interest | -0.03 | 0.07 | 0.77 | 0.09 | 0.05 | 0.24 |
| Political Knowledge | -0.14 | 0.10 | 0.27 | -0.12 | 0.07 | 0.25 |
| Political Efficacy | 0.07 | 0.06 | 0.37 | -0.14 | 0.04 | 0.01* |
| *Political Preferences* |  |  |  |  |  |  |
| Left-Right-Ideology | 0.09 | 0.10 | 0.51 | 0.16 | 0.08 | 0.24 |
| Economic Preferences | 0.13 | 0.09 | 0.25 | 0.09 | 0.07 | 0.36 |
| Cultural Preferences | 0.12 | 0.08 | 0.26 | 0.10 | 0.06 | 0.25 |
| Left-Right-Ideology squared | 0.00 | 0.01 | 0.90 | -0.01 | 0.01 | 0.26 |
| Economic Preferences squared | 0.00 | 0.01 | 0.77 | 0.00 | 0.01 | 0.86 |
| Cultural Preferences squared | 0.00 | 0.01 | 0.90 | 0.01 | 0.00 | 0.25 |
| *Personality* |  |  |  |  |  |  |
| Openness to Experience | 0.00 | 0.06 | 0.98 | -0.06 | 0.04 | 0.25 |
| Neuroticism | 0.01 | 0.06 | 0.93 | 0.11 | 0.04 | 0.06* |
| *Social Media* |  |  |  |  |  |  |
| Frequency of Internet Use | 0.02 | 0.02 | 0.51 | -0.03 | 0.02 | 0.25 |
| Social Media Platforms | -0.05 | 0.04 | 0.25 | 0.01 | 0.02 | 0.77 |
| Political Participation Online | 0.11 | 0.07 | 0.25 | 0.04 | 0.05 | 0.51 |
| *Social & Political Polarization* |  |  |  |  |  |  |
| Social Network Diversity | -0.10 | 0.05 | 0.12 | -0.03 | 0.04 | 0.57 |
| *Populism* |  |  |  |  |  |  |
| Populism Index | 0.87 | 0.08 | 0.00* | 0.84 | 0.06 | 0.00* |
| AfD Identification | 1.28 | 0.19 | 0.00* | 1.22 | 0.21 | 0.00* |
| *Socio-Demographic Controls* |  | *yes* |  |  | *yes* |  |

*Note:* * *q*-values < .1 (i.e. corrected *p*-values that allow for a false discovery rate among significant findings of maximum 10 percent).

Table A3.5: Robustness of M15: Explanations of Subjective Freedom of Speech

|  | | OLS |  | | Logit |  |
| --- | --- | --- | --- | --- | --- | --- |
|  | Est. | SE | q | Est. | SE | q |
| *Social Class* |  |  |  |  |  |  |
| Subjective Social Class | -0.06 | 0.02 | 0.00* | -0.09 | 0.05 | 0.12 |
| *Political Involvement* |  |  |  |  |  |  |
| Political Interest | 0.05 | 0.02 | 0.04* | 0.21 | 0.06 | 0.00* |
| Political Knowledge | -0.07 | 0.03 | 0.07* | 0.03 | 0.09 | 0.76 |
| Political Efficacy | -0.02 | 0.02 | 0.38 | 0.05 | 0.05 | 0.43 |
| *Political Preferences* |  |  |  |  |  |  |
| Left-Right-Ideology | 0.03 | 0.01 | 0.00* | 0.08 | 0.03 | 0.00* |
| Economic Preferences | 0.05 | 0.01 | 0.00* | 0.09 | 0.02 | 0.00* |
| Cultural Preferences | 0.10 | 0.01 | 0.00* | 0.18 | 0.02 | 0.00* |
| *Personality* |  |  |  |  |  |  |
| Openness to Experience | -0.01 | 0.02 | 0.63 | -0.01 | 0.05 | 0.82 |
| Neuroticism | 0.03 | 0.02 | 0.26 | 0.03 | 0.06 | 0.68 |
| *Social Media* |  |  |  |  |  |  |
| Frequency of Internet Use | 0.00 | 0.01 | 0.68 | 0.01 | 0.02 | 0.68 |
| Social Media Platforms | 0.00 | 0.01 | 0.82 | 0.03 | 0.03 | 0.46 |
| Political Participation Online | 0.06 | 0.02 | 0.01* | 0.16 | 0.06 | 0.01* |
| *Social & Political Polarization* |  |  |  |  |  |  |
| Social Network Diversity | -0.04 | 0.02 | 0.02* | -0.06 | 0.04 | 0.31 |
| *Populism* |  |  |  |  |  |  |
| Populism Index | 0.46 | 0.02 | 0.00* | 0.97 | 0.07 | 0.00* |
| AfD Identification | 0.87 | 0.08 | 0.00* | 1.20 | 0.17 | 0.00* |
| *Socio-Demographic Controls* |  | *yes* |  |  | *yes* |  |

*Note:* * *q*-values < .1 (i.e. corrected *p*-values that allow for a false discovery rate among significant findings of maximum 10 percent).

Table A3.6: East/West of M15: Explanations of Subjective Freedom of Speech

|  | | East |  | | West |  |
| --- | --- | --- | --- | --- | --- | --- |
|  | Est. | SE | q | Est. | SE | q |
| *Social Class* |  |  |  |  |  |  |
| Subjective Social Class | -0.09 | 0.05 | 0.16 | -0.12 | 0.04 | 0.01 |
| *Political Involvement* |  |  |  |  |  |  |
| Political Interest | -0.03 | 0.07 | 0.70 | 0.09 | 0.05 | 0.14 |
| Political Knowledge | -0.14 | 0.10 | 0.22 | -0.12 | 0.07 | 0.16 |
| Political Efficacy | 0.07 | 0.06 | 0.34 | -0.13 | 0.04 | 0.00* |
| *Political Preferences* |  |  |  |  |  |  |
| Left-Right-Ideology | 0.11 | 0.03 | 0.00* | 0.04 | 0.02 | 0.14 |
| Economic Preferences | 0.10 | 0.02 | 0.00* | 0.11 | 0.02 | 0.00* |
| Cultural Preferences | 0.13 | 0.02 | 0.00* | 0.19 | 0.02 | 0.00* |
| *Personality* |  |  |  |  |  |  |
| Openness to Experience | 0.00 | 0.06 | 1.00 | -0.06 | 0.04 | 0.22 |
| Neuroticism | 0.01 | 0.06 | 0.93 | 0.11 | 0.04 | 0.03* |
| *Social Media* |  |  |  |  |  |  |
| Frequency of Internet Use | 0.02 | 0.02 | 0.44 | -0.03 | 0.02 | 0.16 |
| Social Media Platforms | -0.05 | 0.04 | 0.19 | 0.01 | 0.02 | 0.68 |
| Political Participation Online | 0.11 | 0.07 | 0.17 | 0.04 | 0.05 | 0.44 |
| *Social & Political Polarization* |  |  |  |  |  |  |
| Social Network Diversity | -0.10 | 0.05 | 0.06* | -0.03 | 0.04 | 0.47 |
| *Populism* |  |  |  |  |  |  |
| Populism Index | 0.87 | 0.08 | 0.00* | 0.85 | 0.06 | 0.00* |
| AfD Identification | 1.29 | 0.19 | 0.00* | 1.24 | 0.21 | 0.00* |
| *Socio-Demographic Controls* |  | *yes* |  |  | *yes* |  |

*Note:* * *q*-values < .1 (i.e. corrected *p*-values that allow for a false discovery rate among significant findings of maximum 10 percent).

Table A3.7: Test of Linearity Assumption

|  | Est. | SE |
| --- | --- | --- |
| M1: Subjective Social Class - 2 | -0.06 | 0.21 |
| M1: Subjective Social Class - 3 | -0.32 | 0.21 |
| M1: Subjective Social Class - 4 | -0.54 | 0.20 |
| M1: Subjective Social Class - 5 | -0.83 | 0.21 |
| M1: Subjective Social Class - 6 | -1.09 | 0.33 |
| M2: Political Interest - 2 | -0.18 | 0.19 |
| M2: Political Interest - 3 | -0.50 | 0.18 |
| M2: Political Interest - 4 | -0.70 | 0.19 |
| M2: Political Interest - 5 | -0.48 | 0.21 |
| M4: Political Efficacy - 2 | -0.19 | 0.16 |
| M4: Political Efficacy - 3 | -0.43 | 0.15 |
| M4: Political Efficacy - 4 | -0.76 | 0.15 |
| M4: Political Efficacy - 5 | -0.75 | 0.16 |
| M9: Frequency of Internet Use - Log | -0.09 | 0.05 |
| M9: Social Media Platforms - Log | -0.07 | 0.07 |
| M9: Political Participation Online - Log | 0.20 | 0.07 |
| *Socio-Demographic Controls* | *yes* |  |

**A.4 Robustness Analysis**

Because of the multitude of hypotheses tests we not only committed ourselves to account for the false discovery rate but also to impose a strict robustness requirement. To be considered robust, effects need to attain statistical significance in three model specifications: the full ordered logit model specification in the previous section, an alternative OLS specification which interprets the subjective freedom of speech item as continuous measure and an alternative logit specification which treats subjective freedom of speech as a dichotomous variable (see table A.3.3 in the online appendix). Only three hypothesized explanatory factors fully survive this strict inference criterion and prove to be completely robust explanations of subjective freedom of speech: political preferences^[[9]](#footnote-9)^, populist attitudes and identification with the AfD. The negative effect of lower subjective social class is only ‘partially robust’ as it reaches statistical significance in the OLS-specification, but not in the logit model. Looking at political involvement, the two previously significant predictors political knowledge and political efficacy lose their significance.^[[10]](#footnote-10)^ The findings for personality traits, diverse discussion networks and social media use (frequency of internet use and number of social media platforms) are robustly *non*significant. Interestingly, active online political participation, which only narrowly missed statistical significance in the main models (q = 0.13), is significantly associated with less subjective freedom in both robustness specifications.

A second robustness analysis is more substantively motivated and considers separate models for East and West Germany. Many East Germans have experienced state repression and censorship under the GDR regime. In addition, even three decades after re-unification many of them might hold political grievances and feel like second-class citizens (see Elff et al. 2022 for multiple contributions on political differences between East and West Germans). As a result, their sense of subjective freedom of speech may not only differ in levels – it is in fact lower, even when controlling for all other socio-economic and political factors addressed in this paper^[[11]](#footnote-11)^ – but also in its underlying determinants. But as the results in tables A.3.4 and A.3.6 (see online appendix) indicate, the three robust explanations for subjective freedom of speech – political preferences, populism, and identification with the AfD – are all significant and equally important predictors of feeling unfree to speak in both parts of the country.^[[12]](#footnote-12)^ This is an important result because it indicates that similar and more general mechanisms are responsible for these explanations of subjective freedom of speech. However, we also observe some differences between East and West Germans. Lower social class, a lack of political efficacy and a neurotic personality only drive lower subjective freedom of speech in the West, but not the East (see A.3.4).

In addition to these two sets of robustness tests, we also probe whether our functional form assumptions have been violated for any of the predictors. These checks have not been registered and as stated in the pre-analysis plan, any failures will not enter the judgement or lead to an adjustment of our hypothesis tests. Instead, we provide them for additional exploratory insight. We test ordered variables (e.g. Likert-scale items) by entering them with dummies for each category (leaving out the first as a reference, see table A.3.7 in the online appendix). Non-linearities in count variables are probed by entering the logged version of these variables. We then check for non-linearities estimating the respective separate models of these variables, controlling for socio-demographics. Results for all ordered variables and most count variables overwhelmingly support the linear functional form assumption. However, logged political participation online proves significant, suggesting that a non-linear functional form specifications might be more accurate in this case. Exact results are provided in table A.3.7 in the online appendix.

**A.5 Additional Exploratory Analyses**

Since political preferences turned out to be a robust and strong driver of a subjective lack of free speech in our analyses, we are curious to learn more about what specific political opinions those who say they cannot speak freely hold. To this end, we related a total of eleven additional political attitude items to subjective freedom of speech (see figure A1).^[[13]](#footnote-13)^ These include attitudes toward specific economic policies, EU integration, climate change policy, gender equality policies, and measures to fight the Covid-19 pandemic (see table A.2.3 in the online appendix for a variable description). Although all these items tap into legitimate policy preferences which clearly have a place in democratic discourse, the holders of these preferences differ markedly in their subjective sense of being able to voice them.

*Figure A1: Exploration: Political Attitudes and Subjective Freedom of Speech. Results from an ordered logit model which controls for socio-demographics.*

Those who are more likely to say they cannot express their opinions freely in public are more likely to think that immigrants should be required to assimilate to German culture, that government measures for gender equality have gone too far, that there should be binding referenda at the federal level, and to a somewhat weaker extent, that the state should stay out of the economy. In contrast, those who are more likely to feel free to express their opinion in public are more likely to prefer further EU integration, think that emergencies like the pandemic justify the restriction of civil liberties and are in favor of increased taxes on fossil fuels to combat climate change. Finally, political opinions about gender quotas in large companies, redistribution, a nation-wide rent cap and mandatory vaccines are not considered particularly costly or related to subjective freedom of speech.

To understand whether the free expression of these eleven political opinions is related to their societal popularity, their degree of societal consensus or their degree of societal polarization, we further correlated the items’ coefficients estimates with the items’ respective means (r = -.26), standard deviation (r = -.33) and kurtosis (a measure of bi-modality, r = .11). However, none of these correlations reveal any strong or significant patterns.

Figure A2 further explores which elements of the populist mindset predict subjective freedom of speech the most by including all six items comprising the populism index *separately* in a joint model. We indeed find that some sub-dimensions of populism are more strongly associated with the sentiment of not being allowed to speak freely than others. The strongest relation is for an item that taps into anti-pluralism and states that compromise in politics is a matter of weak principles. Interestingly, the pro-sovereignty sub-dimension yields mixed results, where one item (‘politicians need to follow the will of the people’) is related to more and a second item (‘people, not politicians should make important policy decisions’) to less subjective freedom of speech. Anti-elitism and imagined homogeneity are all associated with a perceived lack of free expression.

*Figure A2: Exploration: Populist Attitudes and Subjective Freedom of Speech. Results from an ordered logit model which controls for socio-demographics.*

*Figure A3: Exploration: Party Identification and Subjective Freedom of Speech. Results from an ordered logit model which controls for socio-demographics. Identification with AfD serves as reference category.*

Because we also found a strong and robust negative effect on subjective free speech for those who identify with the AfD versus those who do not, figure A3 provides a more detailed picture of how idendification with other political parties (or no party identification at all) compares to AfD support (which is the reference category in this particuar model specification). At least three patterns are noteworthy. First, identification with the AfD seems to have a distinct relation to subjective freedom of speech – all other party supporters are markedly less likely to say they cannot speak freely. Second, there is nonetheless considerable variation across the remaining party identifications. FDP supporters, supporters of smaller parties and those without any party identification are more likely to say that they cannot express themselves freely than supporters of the CDU/CSU, The Left or SPD. Third, and strikingly, no one feels more free to express their opinion in public than those who identify with the Green party. Since key socio-demographics are already accounted for, these differences across parties in general and the effect for the Greens in particular, lend further evidence to the notion that some political standpoints are perceived to be more costly than others.

*Figure A4: Exploration: Social Media Platforms and Subjective Freedom of Speech. Results from an ordered logit model which controls for socio-demographics.*

One striking finding in our main analyses was the virtual non-effect of social media use on subjective freedom of speech. Of the three variables that we use to operationalize the social media hypothesis, the number of social media platforms is arguably the most directly related. Yet, we did not find a significant effect in a single model. We anticipated that this issue might arise, because the variable encompasses a large variety of social media platforms regarding their purpose, popularity and internal structure. For example, LinkedIn and Xing are mostly used for job-networking and there the connection to freedom of speech seems somewhat hazy. Additionally platforms like TikTok expose users to content largely based on their algorithm, while Messengers like Snapchat or Instagram allow you to curate your own feed more strongly. Some platforms such as WhatsApp or Facebook are also much more commonly used than more niche Discord or Signal. Lastly, Telegram might depict a special case because it has been extensively co-opted by political actors from center-right to conspiracy-spreading populists and extremists which makes the platform and its connected groups a potential breeding ground for the narrative of limited freedom of speech.

To investigate whether these differences matter for the subjective sense of free expression, we entered each platform as a separate dummy-variable into a model (see figure A4). We find clear differences across platforms’ association with their users’ perception of free speech. Facebook and Telegram is very strongly, TikTok somewhat weaker associated with a perceived lack of freedom of speech. Users of Signal, Twitter, Threema, Discord or Whatsapp are more likely to feel free to express their opinion. This evidence provides little support for the idea that it is the *type* of platform that matters for free speech. Signal, Whatsapp and Telegram are all very similar in their functionality, but exhibit opposing relationships to subjective freedom of speech. Instead, these platform differences may again at least partly reflect the political stances of their prototypical user base. Telegram, for instance, is the platform of choice for populists and more fringe groups such as conspiracy theorists. These groups are more likely to subscribe to and spread the narrative of limited freedom of speech. Importantly, this exploration shows that, once platform-specific differences are taken into account, social media may be more predictive of subjective freedom of speech than envisaged in our main analysis.

*Figure A5: Exploration: Big Five Personality Traits and Subjective Freedom of Speech. Results from an ordered logit model which controls for socio-demographics.*

**A.5 Appendix References**

Benjamini, Y., and Hochberg, Y. (1995). Controlling the false discovery rate: a practical and powerful approach to multiple testing. *Journal of the Royal Statistical Society Series B*, 57: 289–300.

Gelman, A. (2007). Struggles with survey weighting and regression modeling. *Statistical Science* *22*(2): 153-164.

GLES. (2022). GLES Cross-Section 2021, Pre-Election. GESIS Data Archive, Co-logne. Data file version 2.0.0, doi:10.4232/1.13860

Hanmer, M. J., & Kalkan, O. K. (2013). Behind the curve: Clarifying the best approach to calculating predicted probabilities and marginal effects from limited dependent variable models. *American Journal of Political Science* *57*(1): 263-277.

King, G., Tomz, M., & Wittenberg, J. (2000). Making the most of statistical analyses: Improving interpretation and presentation. *American Journal of Political Science* 44: 347-361.

Little, R. J. A. & Rubin, D. B. (1987). *Statistical analysis with missing data.* New York: John Wiley & Sons.

Neumayer, E., & Plümper, T. (2017). *Robustness Tests for Quantitative Research* (Methodological Tools in the Social Sciences). Cambridge: Cambridge University Press.

Petersen, T. (2021, June 16). Eine Mehrheit fühlt sich gegängelt. *Frankfurter Allgemeine Zeitung (FAZ)*. https://www.faz.net/aktuell/politik/inland/allensbach-umfrage-viele-zweifeln-an-meinungsfreiheit-in-deutschland-17390954.html

Schneider, J. (2021, June 19). Meine Meinung! *ZEIT ONLINE*. https://www.zeit.de/kultur/2021-06/meinungsfreiheit-deutschland-allensbach-umfrage-gefuehle/komplettansicht

Van Buuren, S. (2007). Multiple imputation of discrete and continuous data by fully conditional specification. *Statistical Methods in Medical Research* 16: 219–242.

1. For instance, the Allensbach item (Petersen 2021) requires respondents to trade off free expression with caution: “Do you feel that in Germany today you can freely express your political opinion, or is it better to be cautious?" Arguably, these two concepts do not form opposing poles on the same underlying continuum, because one can feel free to speak and still think that it is a good idea to be cautious when doing so (Schneider 2021). [↑](#footnote-ref-1)
2. While these measures of political preferences directly operationalize our pre-registered hypotheses, we also explore a wide range of additional attitudinal items included in the *GLES 2021 Cross-Section Pre-Election Surve*y. These range from attitudes toward measures against Covid-19 over opinions on climate change to respondents’ views on the economy, the EU, feminism and many more. We stress that we did not report fixed prior assumptions but instead aim to empirically identify which specific political preferences (or clusters of political preferences) are held by individuals that perceive a lack of subjective freedom of speech. Importantly, we explicitly flag these additional (and un-registered) analyses as purely exploratory to clearly separate them from the confirmatory analyses. [↑](#footnote-ref-2)
3. We did not register a priori expectations regarding the remaining three personality dimensions extraversion, conscientiousness, and agreeableness. Instead, we explore their relations to subjective freedom of speech while again clearly separating this from the confirmatory analyses. [↑](#footnote-ref-3)
4. We also explore potential differences in the effects of mainstream public platforms (e.g., Twitter or Instagram) versus niche platforms with stronger group-selection functions (e.g., Telegram) and other subgroup differentiations of social media platforms in order to better inform future research. As we had no fixed prior assumptions regarding the varying effects of different platform types, we explicitly flag these additional (and un-registered) analyses as purely exploratory to clearly separate them from the confirmatory analyses. [↑](#footnote-ref-4)
5. Unfortunately, we cannot use the BIK regions because they are excluded from the scientific use files for data protection reasons. Instead, we make use of a variable that distinguishes between rural and urban residency. [↑](#footnote-ref-5)
6. We use the correction $q = p\times m/i$, where *m* is the number of tests and *i* is the rank of the ordered *p*-values. As critical threshold for these so-called *q*-values we set .10, which corresponds to an FDR of 10 percent. [↑](#footnote-ref-6)
7. In any case, by transparently documenting all results we will still be able to communicate a sense of the *degree* of robustness (cf. Neumayer & Plümper 2017). [↑](#footnote-ref-7)
8. While the pre-registered criterion for our hypotheses tests is M16 and the respective robustness checks, we afterwards chose to also re-run model M15 with OLS- and Logistic-Regression specifications (see table A.3.6) and separate for East and West Germany (see table A.3.7) [↑](#footnote-ref-8)
9. In our pre-analysis plan we only announced robustness checks and East/West sub-sample analyses for the full model specification M16. However, we realized that to evaluate the hypothesized linear effect of political preferences (H3), it is sounder to omit the squared term. Therefore, we re-run the same four robustness models using the specification M15 (see A.3.5 and A.3.6) and relate to those results when evaluating the robustness of the political preferences hypothesis. [↑](#footnote-ref-9)
10. Political interest suddenly turns significant in the opposite direction (i.e. more political interest coincides with less subjective freedom of speech). [↑](#footnote-ref-10)
11. According to the full model specification M16, East Germans are still more likely to say they are not allowed to express themselves in public, already controlling for a host of socio-economic and political variables. [↑](#footnote-ref-11)
12. One exception is the general left-right ideology scale which loses its significance in the West German sample. [↑](#footnote-ref-12)
13. All model specifications reported in this section are ordered logit models that only include the exploratory variables and socio-demographic controls (similar to the separate models discussed further above). [↑](#footnote-ref-13)
